# Micronutrients Associated With Anemia in School-age Children and Adolescents 2005–2018: Biomarkers Reflecting Inflammation and Nutritional Determinants of Anemia (BRINDA) Project

**DOI:** 10.1016/j.cdnut.2025.107502

**Published:** 2025-07-05

**Authors:** Rochelle Werner, Hanqi Luo, Lei Liu, Yuqing Wang, Jiaxi Geng, Yi-An Ko, Parminder S Suchdev, Yaw Addo, Zulfiqar Ahmed Bhutta, Victor Temple, Frank Wieringa, Fabian Rohner, Maria J Ramirez-Luzuriaga, Reina Engle-Stone, Anne Williams, Melissa F Young, BRINDA Workgroup

**Affiliations:** 1 Hubert Department of Global Health, Rollins School of Public Health, Emory University, Atlanta, Georgia, United States; 2 Department of Biostatistics and Bioinformatics, Rollins School of Public Health, Emory University, Atlanta, Georgia, United States; 3 Division of Nutrition, Physical Activity, and Obesity, Centers for Disease Control and Prevention, Atlanta, Georgia, United States; 4 Department of Pediatrics, School of Medicine, Emory University, Atlanta, Georgia, United States; 5 Institute for Global Health & Development, Aga Khan University, Karachi, Pakistan; 6 Centre for Global Child Health, The Hospital for Sick Children, Toronto, Canada; 7 Division of Basic Medical Sciences, School of Medicine and Health Sciences, University of Papua New Guinea, Port Moresby, Papua New Guinea; 8 French National Research Institute for Sustainable Development, Montpellier, France; 9 Phoenix Epidemiology and Clinical Research Section, National Institute of Diabetes and Digestive and Kidney Diseases, Bethesda, Maryland, United States; 10 Department of Nutrition, Institute for Global Nutrition, UC Davis, Davis, California, United States; 11 GroundWork, Fläsch, Switzerland

**Keywords:** anemia, iron deficiency (ID), micronutrients, body mass index (BMI), Biomarkers Reflecting Inflammation and Nutritional Determinants of Anemia (BRINDA), school-age children, adolescents, youth, nutrition, global

## Abstract

**Background:**

School-age children and adolescents may be at risk of anemia through demands on micronutrients required for growth and maturation.

**Objectives:**

This multicountry analysis examined the burden of anemia in children aged 5–19 y by sex and age category and associations with micronutrient deficiencies, inflammation, and BMI.

**Methods:**

Children aged 5–19 y from surveys in the Biomarkers Reflecting Inflammation and Nutritional Determinants of Anemia (BRINDA) Project were included with hemoglobin, ≥1 micronutrient (iron, vitamin A, folate, vitamin B_12_, or zinc) and inflammation biomarker, and *n* > 100 per survey. Factors with bivariate relationships with anemia (*P* < 0.1) were included in multivariable modified Poisson regression models to examine the attributable burden of anemia.

**Results:**

This analysis included 54,534 children from 17 surveys in 16 countries (16 surveys for 15–19 y; 9 surveys for 10–14 y; 8 surveys for 5–9 y). Median overall anemia prevalence was 16% (range: 5% in Ecuador, United Kingdom, and United States to 59% in Côte d’Ivoire) with the highest burden in 15–19-y-old females (24%). In most surveys, anemia prevalence did not differ by sex for children aged 5–14 y, and median anemia prevalence was lower in children aged 10–14 y (7%) than in those aged 5–9 y (9%) or 15–19 y (22%). In most surveys, higher anemia prevalence was associated (*P* < 0.05) with iron deficiency (15%) [prevalence ratio (PR): 1.6–14.2; 5–9 y, 4/7 surveys; 10–14 y, 6/6 surveys; 15–19 y, 13/14 surveys), vitamin A deficiency (2%) (PR: 1.8–3.0; 5–9 y, 2/2 surveys; 10–14 y, 2/3 surveys; 15–19 y, 2/3 surveys), and inflammation (13%) (PR: 1.4–2.4: 5–9 y, 4/4 surveys; 10–14 y, 2/4 surveys; 15–19 y, 6/8 surveys). Folate, vitamin B_12_, zinc, and BMI had weak, variable associations with anemia.

**Conclusions:**

Iron deficiency and vitamin A deficiency are consistently associated with anemia in school-age children and adolescents, whereas inflammation and other micronutrients had context-dependent associations. This research underscores the importance of examining multiple micronutrients associated with anemia in the context of factors such as country, age, and sex.

## Introduction

Anemia is a condition characterized by insufficient oxygen-carrying capacity of red blood cells (RBCs), which can impair cognitive and motor development, reduce work capacity, and ultimately lower economic productivity [1,2]. Anemia is particularly concerning among young children, menstruating adolescent females, women of reproductive age, and pregnant women—due to the potential for severe public health consequences such as reduced birth weight, increased preterm birth, and increased risk of maternal and child mortality [[3], [4], [5]]. Older children and adolescents also have increased demands for micronutrients to support growth and sexual maturation during puberty [6] and may be at risk of anemia and its consequences in settings of suboptimal dietary intake [7].

In 2019, the Global Burden of Disease (GBD) study estimated 1.8 billion cases of anemia globally, with the highest prevalence among children aged 5–9 y [8]. In the GBD study, sex-based differences in the prevalence of anemia emerged in adolescence at the age of menarche in girls (10–14 y), and adolescent females aged 15–19 y had a higher burden of anemia than any other age groups within women of reproductive age (15–49 y) [8]. To attain the World Health Assembly and United Nation’s Sustainable Development Goals’ target of addressing nutritional needs of adolescent girls and reducing anemia in women of reproductive age to end malnutrition by 2030 [9], a greater understanding of factors contributing to anemia can inform the development of future actions and more effective interventions.

Anemia may result from direct causes such as micronutrient deficiencies, infection, inflammation, inherited RBC disorders, and gynecologic or obstetric conditions, as well as intermediate and underlying causes, such as socioeconomic or ecological risk factors [4,10]. Although approximately half of the cases of anemia among nonpregnant women of reproductive age are estimated to be amenable to iron supplementation [11], iron-based interventions alone may be inadequate to address the multifactorial causes of anemia. Deficiencies in iron, vitamin A, folate, vitamin B_12_, and zinc are known potential contributors to anemia [12], which may coincide with each other and vary in prevalence by geographic and economic contexts [4,8]. Previous single-country or population studies of school-age children and adolescents have identified sociological and ecological factors associated with anemia, which include age, sex, and wealth index [[13], [14], [15], [16], [17], [18]]. However, few studies of school-age children and adolescents have examined associations of micronutrient deficiencies with anemia [[19], [20], [21]], and more information is needed to compare multiple factors associated with anemia across countries or regional populations.

The Biomarkers Reflecting Inflammation and Nutritional Determinants of Anemia (BRINDA) Project examined the anemia-attributable burden of various micronutrient deficiencies and inflammation using individual-level data from multiple countries. Previous BRINDA research examined factors associated with anemia [22] in preschool-age children [23] (<5 y), women of reproductive age [24], and pregnancy—but not for school-age children or nonpregnant adolescents. Thus, the aims of this analysis were to estimate the prevalence of anemia by severity, sex, and age and to examine key factors associated with anemia—such as inflammation, micronutrient deficiencies, sex, age, or socioeconomic status—in school-age children and adolescents across multiple countries.

## Methods

### Study setting and data availability

**FIGURE 1:**
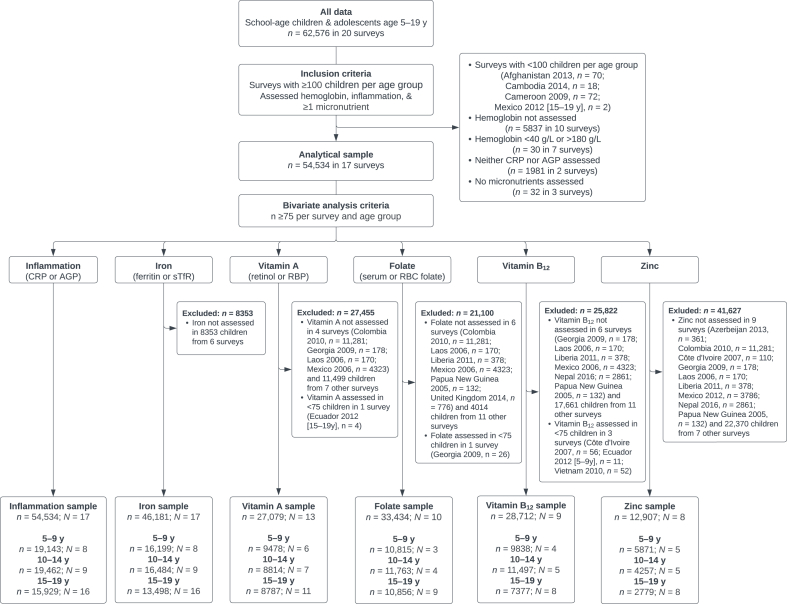
Flow chart of school-age children and adolescents aged 5–19 y included in analysis of inflammation and micronutrients associated with anemia: BRINDA Project. AGP, α-1-acid glycoprotein; BRINDA, Biomarkers Reflecting Inflammation and Nutritional Determinants of Anemia; CRP, C-reactive protein; *N*, number of data sets; *n*, number of children; RBC, red blood cell; RBP, retinol-binding protein; sTfR, soluble transferrin receptor.

The BRINDA Project (www.brinda-nutrition.org) harmonized deidentified data sets of inflammation and micronutrient biomarkers from globally diverse contexts, as described by Namaste et al. [25]. Data sets of school-age children and adolescents were aggregated from national or regional cross-sectional surveys of school-age children, adolescents, and nonpregnant females (15–19 y) included among surveys of women of reproductive age. School-age children and adolescents were selected from data sets in the BRINDA Project based on the following criteria: *1*) age 5–19 y; *2*) measured hemoglobin (Hb), ≥1 biomarker of inflammation [C-reactive protein (CRP) or α-1-acid glycoprotein (AGP)], and ≥1 micronutrient (iron, vitamin A, vitamin B_12_, folate, or zinc); and *3*) sample size of *n* > 100 per survey and age group (5–9 y, 10–14 y, 15–19 y). Of the 20 surveys (*n* = 62,576) available in the BRINDA project, 17 surveys (*n* = 54,534) conducted in 16 countries between 2005 and 2018 met the inclusion criteria (Figure 1). The United States 2018 data set combined survey cycles of National Health and Nutrition Examination Survey (NHANES) data from NHANES 1999–2000 through NHANES 2017–2018. More information on individual survey designs is available online (https://osf.io/mh9rg/files/osfstorage?view_only=1d62f98fa7ec4548976374588b04b8ef). This secondary analysis of deidentified data sets was not determined to be human subject research and was exempted from the institutional review board review.

### Data collection and laboratory methods

Height and weight of 5–19-y-old children were assessed in 15 surveys. Socioeconomic status was assessed using household assets, wealth indices, and poverty to income ratios. Hemoglobin was measured by venous blood in 12 surveys and capillary blood in 5 surveys. CRP or AGP were assessed by ELISA or sandwich ELISA, turbidimetry, immunoturbidimetry, or photometric turbidimetry, nephelometry, or autoanalyzer techniques. Iron biomarkers [serum or plasma ferritin and soluble transferrin receptor (sTfR)] were assessed by ELISA or sandwich ELISA techniques, various chemiluminescence and immunoassays, or photometric turbidimetry. Serum or plasma retinol was assessed by HPLC, and retinol-binding protein (RBP) was assessed by ELISA or sandwich ELISA techniques. Folate biomarkers (serum or plasma folate and RBC folate) and serum or plasma vitamin B_12_ were assessed by microbiological assays, chemiluminescent immunoassays, competitive enzymatic immunoassays, ELISA, or liquid chromatography tandem mass spectrometry. Serum or plasma zinc was assessed by flame atomic absorption spectrophotometry, atomic emission spectrometry, or inductively coupled plasma optical emission or mass spectrometry techniques. Malaria was assessed by microscopy, rapid diagnostic kits, or histidine-rich protein 2 measurement. Survey-specific information on laboratory assessment methods for hemoglobin, inflammation, micronutrient biomarkers, and malaria for school-age children and adolescents is available online.

### Variable definitions

Anemia was defined using hemoglobin concentrations adjusted for altitude and smoking, when available [26]. Hemoglobin values <40 g/L or >180 g/L were deemed biologically implausible and excluded from analysis [27], and any anemia was assessed according to WHO cutoffs for hemoglobin (age <12 y: Hb <115 g/L; age 12–14 y and girls ≥15 y: Hb < 120g/L; boys ≥15 y: Hb < 130 g/L) and further classified among those with anemia as mild if hemoglobin was ≥110 g/L, moderate if hemoglobin was ≥80 g/L and <110 g/L, and severe if hemoglobin was <80 g/L for all age groups [28]. Inflammation was defined by either CRP >5 mg/L or AGP >1 g/L [29,30]. Micronutrient deficiencies were defined using inflammation-adjusted values when applicable according to the following cutoffs: iron deficiency by ferritin <15 μg/L or sTfR >8.3 mg/L, only in absence of measured ferritin [31]; vitamin A deficiency by retinol <0.7 μmol/L or RBP <0.7 μmol/L, only in absence of measured retinol [32]; folate deficiency by serum or plasma folate <10 nmol/L or RBC folate <340 nmol/L, only in absence of serum or plasma folate [33]; vitamin B_12_ deficiency by serum vitamin B_12_ <150 pmol/L [34]; and zinc deficiency by serum or plasma concentrations below cutoffs based on age, sex, fasting status, and time of blood draw recommended by the International Zinc Nutrition Consultative Group [35]. In the absence of guidance for zinc cutoff values for children under 10 y in a fasted state, for morning blood draws we used morning fasting cutoffs for children aged ≥10 y, and for afternoon blood draws we used morning nonfasting cutoffs for children aged <10 y. For children aged ≥10 y with afternoon blood draws in a fasted state, we used morning fasting cutoffs for children aged ≥10 y. Additionally, children with unknown fasting status or time of blood draw were assumed to be nonfasting or have afternoon blood draws.

### Statistical analysis

All analyses were conducted separately by survey in R Studio v 4.2.1 with 2-sided hypothesis testing and statistical significance set to α = 0.05. The analysis was replicated independently by a second data analyst. All results are stratified by age group (5–9 y, 10–14 y, and 15–19 y). Prevalence estimates for anemia, inflammation, and micronutrient deficiencies were assessed separately for each age group and sex as well as in combination.

Micronutrient, CRP, and AGP biomarkers with values of zero or below the limit of detection were replaced with half of the minimum observed value for running regression models on the log-transformed scale. Subsequently, micronutrient biomarkers were adjusted for inflammation using the linear regression approach from the BRINDA R package [25,36,37], with adjustments modeled separately for each survey and combination of available micronutrients, inflammation biomarkers, and age group categories (5–9 y, 10–14 y, and 15–19 y). Briefly, ferritin and sTfR were adjusted for inflammation in all age groups; however, sTfR was adjusted only for AGP, whereas ferritin was adjusted for both CRP and AGP when available [11,36,38]. Retinol and RBP were adjusted for both CRP and AGP, when available, for children 5–9 y and 10–14 y but not 15–19 y [11,36,39]. Two children were excluded from iron and vitamin A analyses because BRINDA inflammation–adjustment method could not be applied: one child was missing CRP and the other was missing AGP, but all other children from their survey and age group had both CRP and AGP. Inflammation adjustment was not indicated for folate, RBC folate, vitamin B_12_, and zinc in school-age children or adolescents [40,41].

The age of 19-y-old adolescents (228.0–239.9 mo) was set to a maximum of 228 mo per requirements of the WHO Anthro Plus package in R [42]. Height and weight were evaluated for biologically plausible *z*-scores: height was set to missing when height-for-age *z*-scores were >6 or <−6, and subsequently weight was set to missing for children aged 5–10 y when weight-for-age *z*-scores were >5 or <−6 and for adolescents 10–19 y when BMI *z*-scores were >5 or <−5. BMI categories were defined by the following BMI *z*-scores: thin (≤−2), normal (>−2 and <1), overweight (≥1 and <2), and obese (≥2) [43].

Inflammation, micronutrient deficiencies, sex, age (continuous and categorical), and socioeconomic status [44] were assessed for bivariate relationships with anemia by modified Poisson regression. Multivariable models were constructed with factors that had bivariate associations of *P* < 0.1 with anemia. These models included micronutrient biomarkers assessed in ≥75 children per survey to allow for some missingness in surveys of small sample size and micronutrients that were assessed only in a subset of children. Additionally, some micronutrients that had bivariate associations with anemia were excluded from multivariable models due to a large reduction (>90%) in sample size or low prevalence of deficiency that prevented model convergence. We used modified Poisson regression with robust standard errors to obtain prevalence ratio (PR) estimates [22] for inflammation, and each micronutrient deficiency that met inclusion criteria while controlling for sex, age, and socioeconomic status when associated with anemia. We compared estimated PRs and confidence intervals (CIs) of associations to those obtained from always controlling for sex, age, and socioeconomic status and for models that considered malaria, inflammation-adjusted vitamin A for all age groups, or BMI category. BMI category may be associated with anemia due to undernutrition or inflammation associated with overweight and obesity [45]; it was included in sensitivity analyses as inflammation and micronutrient deficiencies were hypothesized to have more direct, causal relationships with anemia in school-age children and adolescents (Supplemental Figure 1). Population attributable fractions were estimated from the PRs derived from multivariable models and the prevalence of inflammation and micronutrient deficiencies among surveys with sample sizes large enough to produce stable estimates [22]. All results are unweighted as children included in the original datasets were not selected to be representative for their assigned age group.

## Results

**FIGURE 2:**
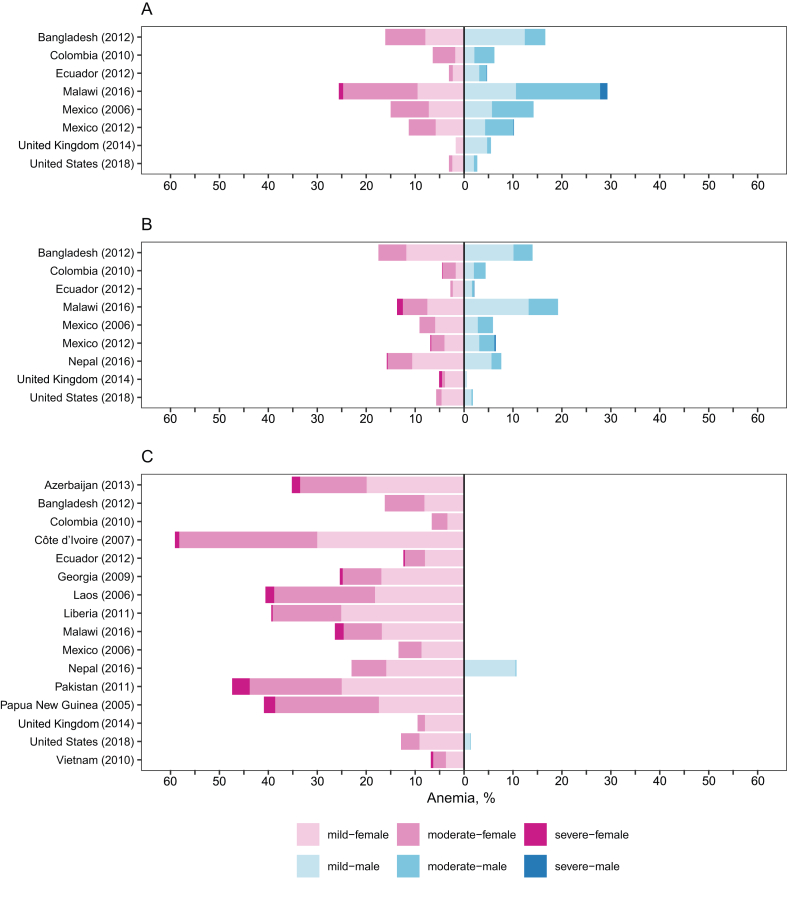
Severity of anemia by sex in children aged (A) 5–9 y, (B) 10–14 y, and (C) 15–19 y in the BRINDA Project. Severity of anemia was assessed using WHO cutoff values for altitude and smoking-adjusted hemoglobin [severe: <80 g/L (all); moderate: ≥80 g/L and <110 g/L (all); mild: ≥110 g/L and <115 g/L (children <12 y), ≥110 g/L and <120 g/L (children 12–14 y or females ≥15 y), ≥110 g/L and <130 g/L (males ≥15 y)] [28]. Only 2 surveys of 15–19-y-old children assessed hemoglobin in males. Sample size for each survey and age group is presented in Table 1, as all children included in analysis had complete measures for hemoglobin and inflammation. BRINDA, Biomarkers Reflecting Inflammation and Nutritional Determinants of Anemia.

This analysis included 17 surveys of school-age children and adolescents aged 5–19 y (*n* = 54,534) (Figure 1). Micronutrient data included in analyses were available in the following number of surveys and children: iron [number of data sets (*N*) = 17; total sample size (*n*) = 46,181), vitamin A (*N* = 13; *n* = 27,079), folate (*N* = 10; *n* = 33,434), vitamin B_12_ (*N* = 9; *n* = 28,712), and zinc (*N* = 8; *n* = 12,907) (Figure 1). Among the 17 surveys included in this analysis, 8 included only 15–19-y-old females, 2 surveys included 15–19-y-old males, and the remaining surveys of 5–14-y-old children included both males and females (Figure 2).

Across surveys, the overall median prevalence of anemia was 16% and ranged from 5% in Ecuador, the United Kingdom, and the United States to 59% in Côte d’Ivoire, with predominantly mild to moderate anemia (Supplemental Table 1). The prevalence of severe anemia was <4% for all countries, with the highest prevalence of severe anemia (3.6%) in Pakistan among 15–19-y-old females (Supplemental Table 1; Figure 2). The median prevalence of anemia was lower in children aged 10–14 y (7%) than children aged 5–9 y (9%) or 15–19 y (22%) (Figure 2). Anemia was similar between females and males of the same survey and age group, with 5 exceptions in which females had higher anemia prevalence: females aged 10–14 y in Nepal, the United Kingdom, and the United States; and females aged 15–19 y in Nepal and the United States (Figure 2). In females, the median values and range of anemia prevalence were overall higher among children aged 15–19 y than those among children aged 5–9 y or 10–14 y. In males, the median anemia prevalence was <10% in all age groups (Figure 2).

The prevalence of inflammation and micronutrient deficiencies combined by age and sex varied widely by survey (Supplemental Table 2) and differed between age categories (Table 1). Median prevalence of inflammation was lowest in children aged 10–14 y, whereas the burden of micronutrient deficiencies was often higher in older adolescents than younger school-age children. The prevalence of iron deficiency was highest in females aged 15–19 y and significantly higher in females than males in 5 of 9 surveys of children aged 10–14 y and 2 of 2 surveys of adolescents aged 15–19 y but not for any of the 8 surveys of children aged 5–9 y (Supplemental Figure 2). Prevalence of inflammation or other micronutrient deficiencies did not differ by sex within an age category apart from 1 of 5 surveys that measured zinc deficiency, in which Ecuadorian males aged 10–14 y had a higher prevalence of zinc deficiency (49%) than females (41%) (data not shown).

**TABLE 1:** Prevalence of inflammation and micronutrient deficiencies in school-age children and adolescents aged 5–19 y by age category: BRINDA Project.

Country survey	Inflammation		Iron deficiency		VA deficiency		Folate deficiency		Vitamin B_12_ deficiency		Zinc deficiency	
	*n*	% (95% CI)	*n*	% (95% CI)	*n*	% (95% CI)	*n*	% (95% CI)	*n*	% (95% CI)	*n*	% (95% CI)
**Children 5–9 y**												
Bangladesh (2012)	638	16.0 (13.3, 18.9)	634	4.6 (3.1, 6.4)	634	21.0 (17.4, 24.8)	—	—	—	—	—	—
Colombia (2010)	4282	14.5 (13.4, 15.6)	4282	8.5 (7.6, 9.4)	—	—	—	—	4009	3.1 (2.6, 3.7)	—	—
Ecuador (2012)	3210	6.7 (5.7, 7.7)	3210	2.3 (1.7, 3.0)	3193	10.6 (8.9, 12.3)	3209	0	—	—	3209	17.7 (16.1, 19.4)
Malawi (2016)	409	44.3 (38.9, 49.6)	409	4.2 (2.4, 6.6)	409	5.1 (3.0, 8.0)	—	—	—	—	407	57.5 (49.5, 65.3)
Mexico (2006)	2428	10.1 (8.9, 11.3)	2428	27.6 (25.6, 29.6)	—	—	—	—	—	—	1917	15.8 (13.9, 17.9)
Mexico (2012)	2681	9.0 (8.0, 10.1)	2680	14.2 (12.9, 15.5)	1945	1.7 (1.2, 2.4)	2679	0.0 (0.0, 0.2)	2680	0.3 (0.2, 0.6)	—	—
United Kingdom (2014)	244	3.3 (1.5, 6.0)	239	17.6 (13.0, 22.9)	223	1.8 (0.6, 4.2)	—	—	232	0	142	0.7 (0.0, 3.1)
United States (2018)	5251	5.9 (5.3, 6.6)	2317	6.3 (5.3, 7.3)	3074	0.8 (0.5, 1.2)	4927	0.1 (0.0, 0.2)	2917	0.1 (0.0, 0.2)	196	2.6 (0.6, 6.6)
**Median (range)**^1^	**—**	**9.6 (3.3–44.3)**	**—**	**7.4 (2.3–27.6)**	**—**	**3.4 (0.8–21.0)**	**—**	**0 (0.0–0.1)**	**—**	**0.2 (0.0–3.1)**	**—**	**15.8 (0.7–57.5)**
**Children 10–14 y**												
Bangladesh (2012)	621	11.6 (9.2, 14.3)	616	7.0 (5.1, 9.2)	614	19.4 (15.9, 23.3)	—	—	—	—	—	—
Colombia (2010)	4310	11.8 (10.7, 12.9)	4310	12.6 (11.6, 13.7)	—	—	—	—	2830	4.7 (4.0, 5.6)	—	—
Ecuador (2012)	2889	5.8 (4.9, 6.9)	2888	4.1 (3.3, 4.9)	88	8.0 (3.8, 14.2)	2889	0.2 (0.1, 0.5)	2800	0.8 (0.4, 1.4)	2886	44.8 (42.8, 46.9)
Malawi (2016)	342	23.7 (19.0, 28.9)	342	3.5 (1.9, 5.9)	342	1.8 (0.7, 3.5)	—	—	—	—	339	57.2 (49.7, 64.5)
Mexico (2006)	1230	8.8 (7.2, 10.5)	1230	22.0 (19.6, 24.6)	—	—	—	—	—	—	482	16.4 (12.9, 20.3)
Mexico (2012)	1105	8.4 (6.8, 10.2)	1105	15.0 (13.0, 17.2)	828	1.1 (0.5, 2.0)	1104	0.2 (0.0, 0.6)	1104	0.6 (0.3, 1.2)	—	—
Nepal (2016)	1588	6.1 (5.0, 7.4)	1588	12.2 (10.3, 14.3)	1588	3.0 (2.1, 4.1)	996	23.4 (20.1, 26.9)	—	—	—	—
United Kingdom (2014)	333	3.6 (1.9, 6.0)	332	14.8 (11.1, 19.0)	318	0.9 (0.2, 2.4)	—	—	320	1.2 (0.4, 2.9)	304	3.6 (1.9, 6.1)
United States (2018)	7044	6.2 (5.7, 6.8)	4071	12.7 (11.5, 13.8)	5034	0.2 (0.1, 0.4)	6774	0.1 (0.0, 0.2)	4443	0.2 (0.1, 0.3)	246	3.7 (1.5, 7.1)
**Median (range)**^1^	**—**	**8.4 (3.6–23.7)**	**—**	**12.6 (3.5–22.0)**	**—**	**1.8 (0.2–19.4)**	**—**	**0.2 (0.1–23.4)**	**—**	**0.8 (0.2–4.7)**	**—**	**16.4 (3.6–57.2)**
**Children 15–19 y**												
Azerbaijan (2013)	361	20.5 (16.4, 25.0)	361	38.2 (33.0, 43.6)	361	1.1 (0.2, 3.0)	345	38.3 (32.9, 43.8)	173	22.0 (15.7, 29.2)	—	—
Bangladesh (2012)	173	13.3 (8.8, 18.9)	167	16.8 (12.0, 22.4)	173	12.1 (7.5, 18.2)	160	42.5 (34.4, 50.8)	164	2.4 (0.8, 5.6)	146	21.2 (15.1, 28.4)
Colombia (2010)	2689	15.2 (13.7, 16.7)	2689	24.2 (22.6, 25.9)	—	—	—	—	169	2.4 (0.7, 5.4)	—	—
Côte d’Ivoire (2007)	110	34.5 (26.4, 43.3)	110	16.4 (10.8, 23.2)	110	0	106	87.7 (79.5, 93.7)	56	19.6 (10.3, 32.0)	—	—
Ecuador (2012)	1263	8.9 (7.5, 10.5)	1263	18.1 (16.1, 20.3)	—	—	1263	1.8 (1.3, 2.5)	1263	1.3 (0.5, 2.6)	1246	49.4 (46.5, 52.4)
Georgia (2009)	178	12.4 (7.7, 18.3)	178	0	—	—	26	38.5 (20.1, 59.4)	—	—	—	—
Laos (2006)	170	12.9 (8.8, 18.0)	170	42.9 (34.4, 51.8)	—	—	—	—	—	—	—	—
Liberia (2011)	378	18.0 (14.1, 22.4)	378	38.6 (33.4, 44.0)	378	1.3 (0.4, 3.2)	—	—	—	—	—	—
Malawi (2016)	167	18.6 (13.6, 24.4)	167	21.0 (15.1, 27.8)	167	1.8 (0.5, 4.5)	160	18.8 (12.7, 26.0)	160	11.2 (6.4, 17.7)	166	58.4 (50.1, 66.5)
Mexico (2006)	665	11.0 (8.8, 13.5)	665	30.2 (26.7, 33.9)	—	—	—	—	—	—	541	17.2 (13.6, 21.2)
Nepal (2016)	1273	7.3 (5.8, 9.0)	1273	18.9 (16.6, 21.2)	1273	1.3 (0.7, 2.0)	842	25.5 (21.8, 29.5)	—	—	—	—
Pakistan (2011)	112	19.6 (13.2, 27.4)	99	42.4 (32.9, 52.4)	84	40.5 (30.6, 50.9)	96	61.5 (51.2, 71.1)	97	55.7 (45.8, 65.2)	79	40.5 (29.7, 52.0)
Papua New Guinea (2005)	132	31.1 (22.7, 40.4)	132	15.2 (9.3, 22.5)	132	0	—	—	—	—	—	—
United Kingdom (2014)	199	10.6 (6.7, 15.5)	196	33.2 (27.1, 39.6)	188	0	—	—	196	8.2 (4.9, 12.5)	185	8.1 (4.6, 12.9)
United States (2018)	7868	9.5 (8.8, 10.2)	5459	18.6 (17.5, 19.9)	5737	0.3 (0.2, 0.5)	7707	0.5 (0.3, 0.6)	5155	1.2 (0.9, 1.6)	225	8.0 (4.6, 12.7)
Vietnam (2010)	191	4.7 (2.3, 8.4)	191	16.2 (11.6, 21.7)	184	2.2 (0.7, 4.8)	177	13.0 (8.3, 18.9)	52	5.8 (0.9, 17.5)	191	38.7 (31.0, 46.9)
**Median (range)**^1^	**—**	**13.1 (4.7–34.5)**	**—**	**20.0 (0.0–42.9)**	**—**	**1.3 (0.0–40.5)**	**—**	**31.9 (0.5–87.7)**	**—**	**7.0 (1.2–55.7)**	**—**	**30.0 (8.0–58.4)**

BRINDA inflammation–adjustment method applied for iron (children 5–19 y) and vitamin A (children 5–14 y) [11,36,38,39]. Inflammation was defined by CRP >5 mg/L or AGP >1 g/L. Micronutrient deficiencies were defined by the following: iron, ferritin <15 μg/L or sTfR >8.3 mg/L (Papua New Guinea); vitamin A, retinol <0.7 μmol/L or RBP <0.7 μmol/L (Azerbaijan, Côte d’Ivoire, Liberia, Malawi, Nepal, and Papua New Guinea); folate, serum or plasma folate <10 nmol/L or RBC folate <340 nmol/L (Nepal); vitamin B_12_, serum vitamin B_12_ <150 pmol/L; zinc, serum or plasma concentrations below the IZiNCG 2012 cutoffs.

Abbreviations: AGP, α-1-acid glycoprotein; BRINDA, Biomarkers Reflecting Inflammation and Nutritional Determinants of Anemia; CRP, C-reactive protein; IZiNCG, International Zinc Nutrition Consultative Group; RBC, red blood cell; RBP, retinol-binding protein; sTfR, soluble transferrin receptor; VA, vitamin A.

1 Median (range) reports the unweighted median, lowest, and highest prevalence across surveys for each age group.

Unadjusted, bivariate associations of inflammation, micronutrients, and sociodemographic variables with anemia are presented in Table 2 and Supplemental Table 3 by survey and age category. Associations of anemia with inflammation varied by age category. Inflammation had bivariate associations with anemia in children aged 5–9 y (4/8 surveys) and 15–19 y (6/16 surveys), but in children aged 10–14 y, associations were significant only in 1 of 8 surveys. Iron deficiency was associated with anemia in children of all age categories (5–9 y: 5/8 surveys; 10–14 y: 6/9 surveys; 15–19 y: 13/15 surveys). Vitamin A deficiency was associated with anemia in approximately half of all surveys with no differences by age category (5–9 y: 2/5 surveys; 10–14 y: 3/5 surveys; 15–19 y: 3/7 surveys). Folate deficiency was associated with anemia in 3 of 9 surveys of children aged 15–19 y and rarely assessed in younger children. Vitamin B_12_ was infrequently associated with anemia (5–9 y: 1/2 surveys; 10–14 y: 1/4 surveys; 15–19 y: 1/6 surveys), and zinc deficiency was associated with anemia in 3 of 4 surveys of children aged 5–9 y but less frequently in other age groups (Table 2). The direction and significance of associations between age and anemia differed by age category: a 1-y increase in age was associated (*P* < 0.05) with lower anemia prevalence in children aged 5–9 y (7/8 surveys) and higher anemia prevalence in those aged 10–14 y (4/9 surveys) but rarely associated in children aged 15–19 y (1/16 surveys) (Supplemental Table 3). Lower socioeconomic status was associated (*P* < 0.05) with anemia in children aged 5–9 y (4/8 surveys) but seldom in children aged 10–14 y (2/9 surveys) or 15–19 y (2/14 surveys) and only in Colombia and the United States for adolescents aged ≥10 y (Supplemental Table 3).

**TABLE 2:** Prevalence ratios of bivariate associations between anemia and inflammation and micronutrient deficiencies among school-age children and adolescents aged 5–19 y: BRINDA Project.

Country survey	Inflammation (95% CI)	Micronutrient deficiencies (95% CI)					
		Iron	Vitamin A	Folate	Vitamin B_12_	Zinc	
**Children 5–9 y**							
Bangladesh (2012)	1.25 (0.81, 1.94)	2.00 (1.13, 3.55)∗	1.26 (0.84, 1.87)	—	—	—	
Colombia (2010)	1.44 (1.08, 1.92)∗	2.75 (2.09, 3.62)∗∗∗	—	—	1.94 (1.17, 3.23)∗	—	
Ecuador (2012)	2.19 (1.34, 3.59)∗∗	3.33 (1.76, 6.31)∗∗∗	2.12 (1.39, 3.24)∗∗∗	—	—	1.88 (1.29, 2.75)∗∗∗	
Malawi (2016)	2.36 (1.68, 3.30)∗∗∗	1.77 (1.04, 3.02)∗	0.34 (0.09, 1.27)	—	—	1.61 (1.13, 2.28)∗∗	
Mexico (2006)	0.85 (0.60, 1.20)	1.20 (0.97, 1.47)	—	—	—	1.19 (0.90, 1.57)	
Mexico (2012)	0.89 (0.59, 1.33)	1.28 (0.96, 1.71)	2.63 (1.48, 4.69)∗∗	—	2.09 (0.61, 7.13)	—	
United Kingdom (2014)	3.69 (0.52, 26.18)	4.69 (1.22, 18.06)∗	—	—	—	—	
United States (2018)	2.10 (1.30, 3.38)∗∗	1.82 (0.89, 3.71)	1.45 (0.21, 10.02)	11.48 (2.30, 57.34)∗∗	—	7.64 (1.08, 53.97)∗	
**Children 10–14 y**							
Bangladesh (2012)	1.17 (0.69, 1.98)	2.47 (1.56, 3.89)∗∗∗	2.08 (1.43, 3.02)∗∗∗	—	—	—	
Colombia (2010)	1.43 (0.99, 2.08)	2.76 (2.05, 3.73)∗∗∗	—	—	2.18 (1.23, 3.85)∗∗	—	
Ecuador (2012)	1.49 (0.65, 3.38)	8.58 (5.24, 14.03)∗∗∗	—	—	3.50 (0.91, 13.43)	1.59 (1.00, 2.53)∗	
Malawi (2016)	1.61 (0.99, 2.63)	1.53 (0.56, 4.20)	1.00 (0.16, 6.10)	—	—	0.80 (0.50, 1.30)	
Mexico (2006)	1.67 (0.99, 2.84)	1.23 (0.80, 1.88)	—	—	—	1.39 (0.58, 3.32)	
Mexico (2012)	1.70 (0.90, 3.20)	1.09 (0.60, 1.99)	1.65 (0.26, 10.70)	—	2.15 (0.34, 13.37)	—	
Nepal (2016)	1.40 (0.89, 2.21)	2.54 (1.93, 3.34)∗∗∗	3.00 (2.00, 4.48)∗∗∗	1.30 (0.95, 1.78)	—	—	
United Kingdom (2014)	3.34 (0.45, 24.72)	7.22 (2.00, 26.00)∗∗	—	—	—	—	
United States (2018)	2.05 (1.44, 2.93)∗∗∗	5.54 (4.28, 7.16)∗∗∗	7.21 (2.72, 19.12)∗∗∗	4.37 (0.73, 26.27)	3.55 (0.56, 22.38)	—	
**Children 15–19 y**							
Azerbaijan (2013)	1.00 (0.71, 1.41)	2.75 (2.06, 3.68)∗∗∗	1.43 (0.53, 3.85)	1.37 (1.03, 1.82)∗	1.04 (0.64, 1.67)	—	
Bangladesh (2012)	1.78 (0.81, 3.92)	3.21 (1.69, 6.11)∗∗∗	1.21 (0.46, 3.14)	2.71 (1.29, 5.66)∗∗	—	1.09 (0.44, 2.73)	
Colombia (2010)	1.61 (1.15, 2.25)∗∗	2.59 (1.95, 3.43)∗∗∗	—	—	—	—	
Côte d’Ivoire (2007)	1.43 (1.07, 1.93)∗	1.53 (1.16, 2.03)∗∗	—	0.82 (0.55, 1.23)	—	—	
Georgia (2009)	1.77 (0.99, 3.17)	—	—	—	—	—	
Laos (2006)	1.14 (0.69, 1.88)	1.37 (0.95, 1.97)	—	—	—	—	
Liberia (2011)	1.51 (1.16, 1.96)∗∗	2.17 (1.69, 2.79)∗∗∗	1.01 (0.34, 3.00)	—	—	—	
Malawi (2016)	1.29 (0.72, 2.33)	2.61 (1.63, 4.19)∗∗∗	2.60 (1.12, 6.05)∗	1.54 (0.88, 2.70)	0.19 (0.03, 1.32)	1.52 (0.87, 2.66)	
Mexico (2006)	1.64 (1.00, 2.71)	2.96 (2.01, 4.35)∗∗∗	—	—	—	1.29 (0.76, 2.18)	
Nepal (2016)	1.53 (1.08, 2.16)∗	2.78 (2.24, 3.45)∗∗∗	3.03 (1.94, 4.75)∗∗∗	0.85 (0.63, 1.15)	—	—	
Pakistan (2011)	1.07 (0.67, 1.72)	1.30 (0.88, 1.93)	0.86 (0.57, 1.30)	0.83 (0.53, 1.27)	1.39 (0.87, 2.22)	1.06 (0.70, 1.59)	
Papua New Guinea (2005)	1.20 (0.79, 1.84)	2.36 (1.68, 3.31)∗∗∗	—	—	—	—	
United Kingdom (2014)	1.59 (0.50, 5.02)	3.45 (1.42, 8.38)∗∗	—	—	1.32 (0.33, 5.24)	2.27 (0.74, 6.98)	
United States (2018)	2.09 (1.70, 2.57)∗∗∗	6.76 (5.74, 7.97)∗∗∗	8.47 (5.54, 12.96)∗∗∗	1.98 (0.87, 4.48)	3.01 (1.79, 5.05)∗∗∗	1.82 (0.59, 5.56)	
Vietnam (2010)	1.69 (0.24, 11.64)	17.20 (5.00, 59.14)∗∗∗	—	5.58 (1.85, 16.86)∗∗	—	5.27 (1.49, 18.58)∗	

Values are prevalence ratios (95% CI) of bivariate associations from unadjusted models. BRINDA inflammation–adjustment method applied for iron (children 5–19 y) and vitamin A (children 5–14 y) [11,36,38,39]. Inflammation was defined by CRP >5 mg/L or AGP >1 g/L. Micronutrient deficiencies were defined by the following: iron, ferritin <15 μg/L or sTfR >8.3 mg/L (Papua New Guinea); vitamin A, retinol <0.7 μmol/L or RBP <0.7 μmol/L (Azerbaijan, Côte d’Ivoire, Liberia, Malawi, Nepal, and Papua New Guinea); folate, serum or plasma folate <10 nmol/L or RBC folate <340 nmol/L (Nepal); vitamin B_12_, serum vitamin B_12_ <150 pmol/L; zinc, serum or plasma concentrations below IZiNCG 2012 cutoffs. The associations between vitamin A and anemia were missing in the United Kingdom and Vietnam because none of the children with vitamin A deficiency (*n* = 7, United Kingdom; *n* = 4, Vietnam) had anemia. Prevalence ratios that differ from the null hypothesis value of 1 are marked by the following: ∗*P* < 0.05; ∗∗*P* < 0.01; ∗∗∗*P* < 0.001.

Abbreviations: AGP, α-1-acid glycoprotein; BRINDA, Biomarkers Reflecting Inflammation and Nutritional Determinants of Anemia; CRP, C-reactive protein; IZiNCG, International Zinc Nutrition Consultative Group; RBC, red blood cell; RBP, retinol-binding protein; sTfR, soluble transferrin receptor.

Among children aged 5–9 y, age was the most consistent factor associated with anemia in adjusted multivariable models (*P* < 0.05: 7/7 surveys), with PRs ranging from 0.59 (95% CI: 0.38, 0.94) in the United Kingdom to 0.91 (95% CI: 0.84, 1.00) in Colombia (Table 3). Iron deficiency and inflammation were frequently associated with anemia, and vitamin A deficiency was associated with anemia in some surveys. Although children had complete measures for sex, age, inflammation, and iron deficiency across surveys, the factor contributing to the greatest percentage point change in anemia varied by survey for children aged 5–9 y: iron deficiency for 3 surveys (Bangladesh, Colombia, and Ecuador); inflammation for 2 surveys (Malawi and the United States); and age for 2 surveys (Mexico 2006 and the United Kingdom). Iron deficiency was a factor for anemia among children aged 5–9 y in low-and-middle income countries but not for those in high-income countries. Among surveys that assessed vitamin A, vitamin A deficiency had the greatest magnitude of association with anemia only in Mexico 2012. Folate, vitamin B_12_, and zinc deficiency were rarely assessed or associated with anemia in children aged 5–9 y.

**TABLE 3:** Prevalence ratios of factors associated with anemia among school-age children and adolescents aged 5–19 y in multivariate models: BRINDA Project.

Country survey	*n*	Female	Age (y)	SES category				Inflammation and micronutrient deficiencies					
				High	Medium	Low	*P*	Inflammation	Iron	Vitamin A	Folate	Vitamin B_12_	Zinc
**Children 5–9 y**													
Malawi (2016)	407	—	0.90 (0.81, 1.00)∗	REF	1.88 (0.95, 3.73)	2.39 (1.21, 4.73)∗	0.061	2.25 (1.61, 3.15)∗∗∗	1.99 (1.15, 3.45)∗	—	—	—	1.28 (0.91, 1.79)
Ecuador (2012)	3193	0.65 (0.46, 0.92)∗	0.82 (0.72, 0.93)∗∗	REF	0.96 (0.49, 1.86)	1.53 (0.83, 2.81)	0.051	2.08 (1.25, 3.45)∗∗	2.88 (1.50, 5.52)∗∗	1.77 (1.16, 2.72)∗∗	—	—	1.61 (1.08, 2.39)∗
Bangladesh (2012)	634	—	—	REF	0.76 (0.45, 1.30)	1.33 (0.83, 2.13)	0.054	—	1.81 (1.02, 3.21)∗	—	—	—	—
Colombia (2010)	4009	—	0.91 (0.84, 1.00)∗	REF	1.27 (0.58, 2.79)	2.21 (1.04, 4.68)∗	0.001	1.38 (1.02, 1.88)∗	2.74 (2.04, 3.68)∗∗∗	—	—	1.63 (0.99, 2.68)	—
Mexico (2006)	2427	—	0.87 (0.81, 0.94)∗∗∗	REF	1.52 (0.94, 2.47)	1.58 (0.99, 2.53)	0.191	—	1.12 (0.91, 1.38)	—	—	—	—
Mexico (2012)	1944	—	0.81 (0.74, 0.89)∗∗∗	—	—	—	—	—	1.10 (0.77, 1.55)	2.52 (1.47, 4.30)∗∗	—	—	—
United Kingdom (2014)^1^	239	—	0.59 (0.38, 0.94)∗	—	—	—	—	—	3.62 (0.88, 14.85)	—	—	—	—
United States (2018)	5251	—	0.82 (0.73, 0.92)∗∗	—	—	—	—	2.09 (1.30, 3.37)∗∗	—	—	—	—	—
**Children 10–14 y**													
Malawi (2016)	342	—	—	—	—	—	—	1.61 (0.99, 2.63)	—	—	—	—	—
Nepal (2016)	1588	1.87 (1.36, 2.57)∗∗∗	1.12 (1.03, 1.23)∗∗	—	—	—	—	—	2.12 (1.61, 2.79)∗∗∗	2.93 (1.95, 4.40)∗∗∗	—	—	—
Ecuador (2012)	2796	—	1.50 (1.25, 1.80)∗∗∗	—	—	—	—	—	6.49 (3.98, 10.60)∗∗∗	—	—	3.97 (0.99, 15.84)	1.45 (0.93, 2.29)
Bangladesh (2012)	611	—	—	—	—	—	—	—	2.05 (1.29, 3.25)∗∗	1.96 (1.35, 2.86)∗∗∗	—	—	—
Colombia (2010)	2830	—	1.36 (1.16, 1.60)∗∗∗	REF	1.39 (0.50, 3.89)	2.92 (1.12, 7.65)∗	0.002	1.48 (0.94, 2.31)	2.11 (1.40, 3.17)∗∗∗	—	—	1.90 (1.08, 3.33)∗	—
Mexico (2006)	1230	1.34 (0.79, 2.28)	1.10 (0.94, 1.29)	—	—	—	—	1.70 (1.00, 2.89)∗	—	—	—	—	—
Mexico (2012)	1105	—	—	—	—	—	—	—	—	—	—	—	—
United Kingdom (2014)^1^	332	7.27 (0.97, 54.80)	—	—	—	—	—	—	5.59 (1.64, 19.04)∗∗	—	—	—	—
United States (2018)	3199	2.93 (1.84, 4.68)∗∗∗	1.16 (1.02, 1.31)∗	REF	2.07 (1.04, 4.09)∗	2.67 (1.37, 5.22)∗∗	0.012	2.43 (1.64, 3.60)∗∗∗	4.08 (2.98, 5.59)∗∗∗	3.50 (0.52, 23.42)	—	—	—
**Children 15–19 y**													
Liberia (2011)	378	—	—	—	—	—	—	1.66 (1.29, 2.13)∗∗∗	2.26 (1.77, 2.89)∗∗∗	—	—	—	—
Malawi (2016)	160	—	—	—	—	—	—	—	2.04 (1.21, 3.45)∗∗	2.03 (0.53, 7.84)	—	0.22 (0.04, 1.27)	—
Laos (2006)	170	—	—	—	—	—	—	—	1.37 (0.95, 1.96)	—	—	—	—
Georgia (2009)	178	—	—	—	—	—	—	1.77 (0.99, 3.16)	—	—	—	—	—
Papua New Guinea (2005)	132	—	—	—	—	—	—	—	2.36 (1.68, 3.31)∗∗∗	—	—	—	—
Nepal (2016)	1273	1.64 (1.20, 2.25)∗∗	—	—	—	—	—	1.69 (1.20, 2.38)∗∗	2.47 (1.98, 3.10)∗∗∗	2.31 (1.42, 3.76)∗∗	—	—	—
Côte d’Ivoire (2007)	110	—	1.11 (0.99, 1.25)	—	—	—	—	1.47 (1.10, 1.97)∗	1.57 (1.18, 2.10)∗∗	—	—	—	—
Azerbaijan (2013)	345	—	—	—	—	—	—	—	2.69 (1.99, 3.64)∗∗∗	—	1.20 (0.92, 1.58)	—	—
Ecuador (2012)	1245	—	—	REF	1.46 (0.84, 2.53)	1.66 (0.97, 2.84)	0.227	1.24 (0.81, 1.92)	7.04 (5.21, 9.51)∗∗∗	—	—	—	1.45 (1.09, 1.92)∗
Bangladesh (2012)	159	—	—	—	—	—	—	—	2.77 (1.44, 5.32)∗∗	—	2.38 (1.14, 4.97)∗	—	—
Vietnam (2010)	177	—	—	—	—	—	—	—	14.17 (4.30, 46.69)∗∗∗	—	2.79 (1.04, 7.48)∗	—	3.85 (1.17, 12.69)∗
Pakistan (2011)	112	—	—	—	—	—	—	—	—	—	—	—	—
Colombia (2010)	2689	—	—	REF	0.85 (0.50, 1.46)	1.33 (0.82, 2.16)	0.034	1.58 (1.14, 2.21)∗∗	2.58 (1.95, 3.41)∗∗∗	—	—	—	—
Mexico (2006)	665	—	—	—	—	—	—	1.75 (1.08, 2.83)∗	3.00 (2.05, 4.41)∗∗∗	—	—	—	—
United Kingdom (2014)	196	—	—	—	—	—	—	—	3.45 (1.43, 8.36)∗∗	—	—	—	—
United States (2018)	3496	7.08 (3.93, 12.77)∗∗∗	—	REF	1.88 (1.23, 2.87)∗∗	2.00 (1.33, 3.00)∗∗	0.010	1.52 (1.16, 1.98)∗∗	3.89 (3.13, 4.84)∗∗∗	3.00 (2.06, 4.38)∗∗∗	—	1.50 (0.81, 2.80)	—

Values are prevalence ratios (95% CI). SES categories were defined by classifying the highest quintile as “high,” the lowest two quintiles as “low,” and the third and fourth quintile as “medium”, and *P* values for Wald statistics determined overall significance of the SES variable. BRINDA inflammation–adjustment method was applied for iron (children 5–19 y) and vitamin A (children 5–14 y) [11,36,38,39]. Inflammation was defined by CRP >5 mg/L or AGP >1 g/L. Micronutrient deficiencies were defined by the following: iron, ferritin <15 μg/L or sTfR >8.3 mg/L (Papua New Guinea); vitamin A, retinol <0.7 μmol/L or RBP <0.7 μmol/L (Azerbaijan, Côte d’Ivoire, Liberia, Malawi, Nepal, and Papua New Guinea); folate, serum or plasma folate <10 nmol/L or RBC folate <340 nmol/L (Nepal); vitamin B_12_, serum vitamin B_12_ <150 pmol/L; zinc, serum or plasma concentrations below IZiNCG 2012 cutoffs. Multivariable models adjusted for age, sex, and SES when the *P* values for bivariate associations with anemia were <0.1. Inflammation and micronutrient biomarkers were included in models when bivariate associations with anemia were <0.1, except for biomarkers excluded because they were not measured in the same sample as other micronutrients (folate in Bangladesh, vitamin B_12_ in Ecuador, and zinc in the United States). Surveys are listed in order of country GDP.

Abbreviations: AGP, α-1-acid glycoprotein; BRINDA, Biomarkers Reflecting Inflammation and Nutritional Determinants of Anemia; CRP, C-reactive protein; GDP, gross domestic product; IZiNCG, International Zinc Nutrition Consultative Group; REF, reference group; RBC, red blood cell; RBP, retinol-binding protein; SES, socioeconomic status; sTfR, soluble transferrin receptor.

∗*P* < 0.05; ∗∗*P* < 0.01; ∗∗∗*P* < 0.001.

1 Recommend interpreting estimates and significance with caution since fewer than 12 children had anemia in these age-stratified surveys.

Among children aged 10–14 y, iron deficiency was the most consistent factor associated with anemia and was associated with 2.1–6.5 times higher anemia in 6 of 6 surveys in which iron was examined in multivariable models. Age was the second most consistent factor associated with anemia (4/5 surveys), with magnitudes of association ranging from 1.1–1.5 times higher prevalence of anemia for each year of age. Sex, socioeconomic status, inflammation, and vitamin A deficiency each had significant associations with anemia in 2 surveys; vitamin B_12_ deficiency was associated with anemia in 1 survey; and zinc and folate deficiencies were not associated with anemia. Additionally, none of the examined factors were associated with anemia in 2 surveys (Malawi 2016 and Mexico 2012) (Table 3). Factors associated with anemia in children aged 10–14 y did not appear to differ between higher-income and lower-income countries.

Among adolescents aged 15–19 y, iron deficiency was the most consistent factor associated with anemia (13/14 surveys) in multivariable models with magnitudes of association ranging from 1.6–14.2 times higher anemia. Inflammation had the second most consistent association with anemia (6/8 surveys), with 1.5–1.8 times higher anemia prevalence. Sex retained a significant association with anemia for 2 of 2 surveys that assessed males aged 15–19 y, and in the United States, sex was the factor with the highest magnitude of association (PR: 7.1) with anemia. Socioeconomic status, vitamin A deficiency, folate deficiency, and zinc deficiency were each associated with anemia in 2 surveys, whereas age and vitamin B_12_ deficiency were not associated with anemia (Table 3).

**FIGURE 3:**
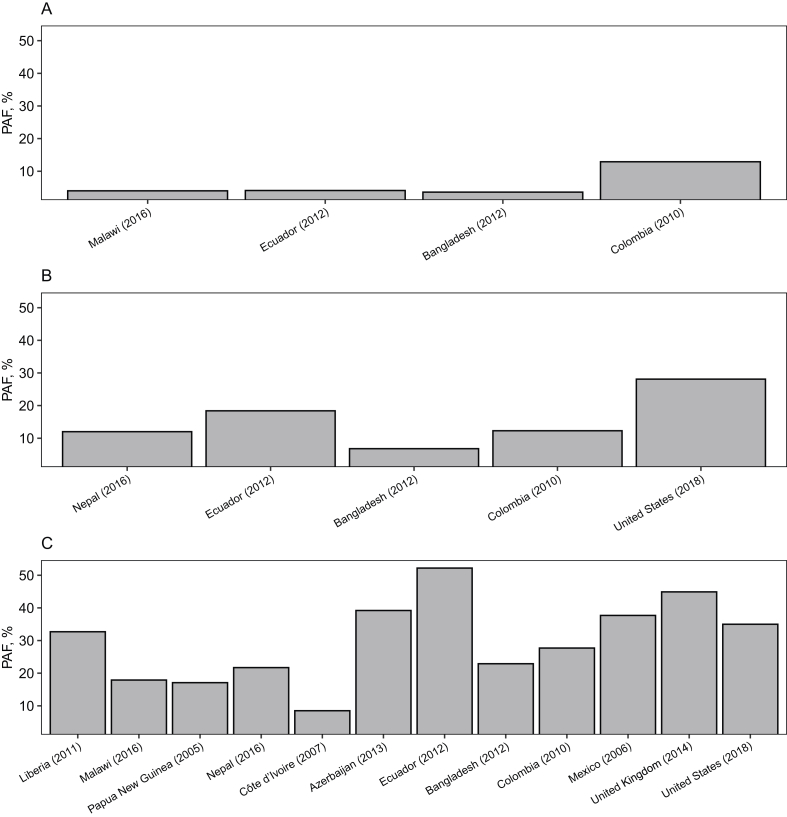
Burden of anemia attributable to iron deficiency in children aged (A) 5–9 y, (B) 10–14 y, and (C) 15–19 y in the BRINDA Project. BRINDA inflammation-adjusted iron deficiency [11,36,38] was assessed using ferritin <15 μmol/L or sTfR >8.3 mg/L when ferritin was unavailable. Population attributable fraction (PAF) was estimated using prevalence ratios [22] from Table 3 and presented in this figure when *P* < 0.05 and estimates were stable. Supplemental Table 4 shows PAF for all micronutrients and inflammation. Sample size for each PAF model is presented in Table 3, and surveys are listed in order of country GDP. BRINDA, Biomarkers Reflecting Inflammation and Nutritional Determinants of Anemia; GDP, gross domestic product; sTfR, soluble transferrin receptor.

The burden of anemia attributable to iron deficiency ranged 4%–13% (median: 4%) in children aged 5–9 y; 7%–41% (median: 15%) in children aged 10–14 y; and 9%–52% (median: 28%) in adolescents aged 15–19 y (Figure 3). Vitamin A was often the micronutrient with the second highest magnitude of association with anemia, and the burden of anemia attributable to vitamin A deficiency was higher than iron among children aged 5–9 y in Ecuador (7.5%) and children aged 10–14 y in Bangladesh (16%). In females aged 15–19 y, the burden of anemia attributable to folate deficiency was 37% in Bangladesh and to zinc deficiency was 18% in Ecuador. In Malawian children aged 5–9 y, the burden of anemia attributable to inflammation was 36%. Population attributable fractions for other micronutrient deficiencies and inflammation were typically <15% and are presented by country and age group in Supplemental Table 4.

For an exploratory analysis, we examined the relationship between BMI category and anemia and added BMI category to the principle multivariable models when *P* < 0.1. Most children had a normal BMI. The prevalence of thinness ranged from 0% (United Kingdom) to 4% (Malawi) in children aged 5–9 y, 1% (United States) to 16% (Nepal) in children aged 10–14 y, and 0% (United Kingdom) to 8% (Vietnam) in adolescents aged 15–19 y. The prevalence of overweight children ranged from 6% (Malawi) to 21% (United States) in children aged 5–9 y, 0% (Malawi) to 27% (United Kingdom) in children aged 10–14 y, and 0% (Vietnam) to 23% (Mexico 2006) in adolescents aged 15–19 y. Prevalence of overweight was similar to the prevalence of obesity, which ranged from 1% (Malawi) to 21% (United States) in children aged 5–9 y, 0% (Malawi) to 26% (United States) in children aged 10–14 y, and 0% (Malawi and Vietnam) to 21% (United States) in adolescents aged 15–19 y. In most surveys, BMI category was not associated (*P* ≥ 0.05) with anemia. Obesity was associated with a 76% lower burden of anemia in children aged 10–14 y in Mexico (2012) (Supplemental Table 5), and BMI category was the only factor examined and associated with anemia in multivariable models for that survey and age category (Supplemental Table 6). In the United States, anemia prevalence was 1.6 times higher in children aged 10–14 y with overweight BMIs and in adolescents aged 15–19 y with obesity than in children of the same age with normal BMIs (Supplemental Table 5). Including BMI category in multivariable models resulted in marginal changes in magnitude of PRs and did not affect the overall interpretation of results (Supplemental Table 6).

The prevalence of malaria (data not shown) varied by survey: 5% in Côte d’Ivoire (15–19 y only), 23% in Liberia (15–19 y only), and 36% in Malawi (5–19 y). Malaria was not associated with anemia in Liberian or Malawian adolescents aged 15–19 y. However, in the remaining models, malaria had the highest magnitude of association among factors examined in multivariate models (PR: 3.11; 95% CI: 2.12, 4.55, in Malawian children aged 5–9 y; PR: 2.99; 95% CI: 1.80, 4.95, in Malawian children aged 10–14 y; and PR: 1.71; 95% CI: 1.29, 2.25, in Côte d’Ivoire adolescents aged 15–19 y) and was the only factor associated with anemia in Malawian children aged 10–14 y.

Adjusting vitamin A for inflammation in adolescents aged 15–19 y reduced the prevalence of vitamin A deficiency from 1.8% to 1.2% in Malawi and from 1.3% to 0.6% in Nepal and thus increased PRs with anemia (4.00; 95% CI: 1.26, 12.74, in Malawi and 2.78; 95% CI: 1.69, 4.56, in Nepal). Model selection and PRs for other variables were unaffected.

## Discussion

This multicountry analysis estimated the burden of anemia attributable to inflammation and micronutrient deficiencies in children aged 5–19 y by sex and age category. Females aged 15–19 y had the highest burden of anemia (median prevalence: 24%; range: 7%–59%). Population attributable fractions and factors associated with anemia differed by age category. Across surveys of children aged 5–9 y, higher burden of anemia was most consistently associated with younger age, followed by inflammation, iron deficiency, and vitamin A deficiency. In surveys of adolescents aged 10–14 y and 15–19 y, iron deficiency had the most consistent and highest magnitude of associations with anemia. Vitamin A was often the micronutrient with the second highest magnitude of association with anemia, whereas folate, vitamin B_12_, and zinc were less frequently assessed in surveys and associated with anemia in some contexts. Factors like inflammation, socioeconomic status, BMI category, and malaria also varied by survey context. Accordingly, the burden of anemia was most frequently attributed to iron deficiency, with higher median population attributable fractions in children aged 15–19 y (28%) than children aged 10–14 y (15%) or 5–9 y (4%). In children aged 5–9 y, anemia was associated with iron deficiency in lower-income countries but not with those in higher-income countries.

Similar to individual surveys included in the BRINDA data sets, other single-country or population studies have reported mixed associations of age and anemia. Anemia prevalence was higher in older than younger adolescents in Nepal [13] and in Ghanaian females [17] but lower in older than younger adolescents in South Ethiopia [16] and in Ghanaian males [17]; it did not differ in Tanzanian adolescents aged 10–17 y [14]. Additionally, anemia was more prevalent among females than males in the studies from Nepal, Ghana, and Tanzania. This analysis included surveys with limited data on males aged 15–19 y. Nevertheless, our findings suggest sex-based differences in the prevalence of anemia or iron deficiency may occur in adolescents aged >10 y but less so for children aged 5–9 y, which is consistent with findings from the GBD study [8]. Among children aged 5–19 y, this study similarly found highest anemia prevalence at age 5–9 y with decreasing prevalence by age for males, whereas in females, anemia was lowest at age 10–14 y and highest at age 15–19 y.

Iron was the most commonly assessed micronutrient deficiency in this set of surveys and among other studies of school-age children and adolescents [46]. In this analysis, iron deficiency was the most consistent and often strongest factor associated with anemia in adolescents aged 10–14 y and 15–19 y, whereas age, inflammation, and iron deficiency were important factors associated with anemia in children aged 5–9 y. However, other single-country analyses that examined micronutrient deficiencies did not indicate a consistent factor with the greatest association with anemia: for example, *1*) in an Indian 2018 nationally representative survey of school-age children (5–9 y) and adolescents (10–19 y), anemia of other causes (no inflammation or deficiencies in iron, folate, or vitamin B_12_) was the leading type of anemia, followed by folate or vitamin B_12_ deficiency, iron deficiency, dimorphic anemia (deficiencies in both iron and folate or vitamin B_12_), and inflammation [19]; *2*) in a Mexico 1999 survey of children aged 0.5–11 y, anemia was mostly associated with iron deficiency but also with folate and vitamin A deficiencies [21]; and *3*) in a Côte d’Ivoire 2010 survey of children aged 6–8 y, anemia had the strongest association with inflammation and secondarily with iron deficiency [20]. These studies used varying age groups and analytical methods to quantify and compare associations within studies, and therefore, the findings are not directly comparable to other studies. In contrast, this analysis examined the burden of anemia attributable to inflammation or multiple micronutrient deficiencies in multiple countries using consistent analytical methods. Nevertheless, comparisons across surveys in this analysis should be interpreted with consideration to the variability in survey-specific data and methods used to assess variables.

Multicountry analyses on etiological factors associated with anemia have been conducted by the BRINDA working group for preschool-age children and women of reproductive age. Similar to findings about preschool-age children [23], anemia in children aged 5–9 y was commonly associated with age, iron deficiency, and inflammation, whereas associations with vitamin A deficiency varied by survey context. Factors associated with anemia in adolescents aged 10–14 y and 15–19 y were similar to those among women of reproductive age: iron deficiency, inflammation, age (of 10–14 y children), vitamin A deficiency, and socioeconomic status were associated with anemia, whereas folate and vitamin B_12_ had few significant associations [24]. Additionally, sex was seldom associated with anemia in children aged 5–9 y or 10–14 y but an important consideration for adolescents aged 15–19 y.

Because we used secondary data, this analysis of school-age children and adolescents was limited to the set of inflammation and micronutrient biomarkers collected by various methods in each survey. Data on males aged 15–19 y were limited to 2 surveys. Children included in the analysis were from surveys that were not designed to be representative for these age groups, and some surveys measured nutrition biomarkers like folate and vitamin B_12_ in women of reproductive age (females aged 15–19 y) but not in younger children or vitamin A only in younger children (<15 y). Accordingly, few studies included in this analysis had complete data for iron, vitamin A, folate, vitamin B_12_, and zinc deficiencies; therefore, it was not possible to compare multivariable models across a consistent set of factors for this analysis. In Nepal, folate deficiency was excluded in multivariable models for children aged 10–14 y: although folate deficiency was 23.4% in this group and had a weak bivariate association with anemia (*P* < 0.1), folate deficiency was assessed only in females. Furthermore, folate deficiency in Nepalese females aged 10–14 y was not significantly associated with anemia when examined in multivariable models with iron and vitamin A. In the United States, folate deficiency was excluded in multivariable models of children of <15 y because models did not converge with the low prevalence of folate deficiency (0.1%). Additionally, zinc deficiency was excluded for all age groups in the United States because it was assessed only in a small subset of children and decreased the model size by >93% (from 3199 to <200 children per age group). Additionally, excluding children with missing data for multivariable analytical models enhances concerns of nonrepresentative samples of children within surveys. Micronutrients, inflammation, and sociodemographic variables included in multivariable models differed across surveys based on strength of association with anemia in bivariate models, and exclusion of potential confounders could affect the results.

Biomarkers used to assess inflammation and deficiencies in iron, vitamin A, and folate varied by survey as did the blood source, analytical laboratory methods, and quality controls for all inflammation and micronutrient biomarkers, including hemoglobin. Surveys differed in whether iron and vitamin A biomarkers were adjusted for inflammation using CRP only or both CRP and AGP. To mitigate the impact of drawing inferences from heterogeneous data sources, models were constructed separately by age group and survey and then compared across contexts qualitatively instead of using pooled estimates. The population attributable fractions in this analysis combined information regarding the prevalence of inflammation and micronutrient deficiencies with their strength of association for anemia. Since all surveys were cross-sectional, the population attributable fractions do not imply that inflammation or micronutrient deficiencies preceded, caused, or could treat anemia [22]. Lastly, population attributable fractions in this analysis may not be generalizable to their respective countries because the original surveys and analysis were not designed or conducted to be representative of school-age children and adolescents.

Importantly, the surveys in the BRINDA database had limited or no information concerning intermediate and underlying determinants of anemia, including infectious diseases, genetic conditions, maturation indicators, and menstruation [47] or gynecologic history of menarcheal females. Anemia and water treatment, sanitation, and hygiene practices have been examined previously in the data sets included in the BRINDA database; however, they were not included in this analysis that specifically focused on associations of micronutrients with anemia. Inclusion of such factors could potentially explain why anemia was not associated with inflammation or any micronutrients in a few surveys or change the estimated PRs of associations with anemia by reducing residual confounding. Lastly, stratifying results and examining models under multiple assumptions created numerous tests, and the threshold for significance (*P* < 0.05) was not adjusted for multiple testing.

This multicountry analysis used existing survey data to address knowledge gaps concerning the anemia-attributable burden of inflammation and micronutrient deficiencies in school-age children and adolescents. This study contributes key information to bridge data gaps in anemia and micronutrient deficiencies across the life cycle. Although selected nutritional biomarkers and analytical methods were heterogeneous, variables across surveys were harmonized according to standardized definitions and cutoff values, adjusted for inflammation by age group according to the BRINDA recommendations for school-age children and adolescents, and analyzed by the same methods and inclusion criteria for all surveys. This facilitated the comparison of data across surveys in varied contexts, including age range and sex of children. Descriptive statistics for anemia were reported separately by age group and sex, and micronutrient deficiencies were examined for sex-based differences within age group–specific models. By examining the prevalence of anemia in multivariable models, this study provided data on the burden of anemia associated with inflammation and micronutrient deficiencies to advance understanding of the multifactorial etiologies of anemia by age group in multiple countries.

Understanding the factors associated with anemia and comparing associations between potentially overlapping micronutrient deficiencies are important for developing actions designed to inform public health strategies to prevent anemia. Although past initiatives have typically targeted the reduction of anemia among women of reproductive age and preschool-age children [10], the narrative is shifting toward integrating programs and initiatives across the lifespan—with particular attention to investing in nutrition, health, and education of adolescents as a means of breaking the intergenerational cycle of malnutrition, including anemia [6,48]. With the present emphasis on adolescent females, little information is available or known about the health of adolescent males [49]. Moreover, integrating programs and services across the lifespan requires a comprehensive understanding of normative developmental stages and nutritional needs for boys and girls aged 5–19 y [6] and the unique contextual factors or barriers they may experience [7]. Treating this age range of children as one homogeneous group could overlook important biological and socioenvironmental differences that may impact nutritional assessment and the effectiveness of programs or interventions. Similarly, approaches to address anemia through single micronutrient interventions may not be effective in contexts with multiple co-occurring micronutrient deficiencies or inflammation [50,51]. This could potentially explain the mixed findings in the limited number of studies examining the effects of micronutrient supplements containing iron only, iron and folic acid, and multiple micronutrients on anemia in school-age children and adolescents [6,49]. Furthermore, standardizing and integrating biological and socioecological indicators in survey designs—and sharing deidentified participant data—may help advance understanding of factors associated with anemia and inform more contextualized approaches to reduce the burden of anemia [10].

## Author contributions

The authors’ responsibilities were as follows – ERW, MFY, HL, YK, PSS: designed the research; ERW: performed the statistical analyses; ERW, MFY: wrote the manuscript; ZAB, VT, FW, FR, MJR-L, RE-S, AW: provided data sets; ERW, HL, LL, YW, JG, YK, PSS, OYA, ZAB, VT, FW, FR, MJR-L, RE-S, AW, MFY: reviewed and edited manuscript; ERW, MFY: had primary responsibility for final content; and all authors: have read and approved the final manuscript.

## Data availability

Data described in the manuscript, code book, and analytic code will be made available upon request pending approval from the BRINDA steering committee and country survey representative.

## Funding

This BRINDA study was supported by the Bill & Melinda Gates Foundation, Centers for Disease Control and Prevention, *Eunice Kennedy Shriver* National Institute of Child Health and Human Development, HarvestPlus, and the United States Agency for International Development. The funders did not influence the design, implementation, analysis, or interpretation of the data. The findings and conclusions in this report are those of the authors and do not necessarily represent the official position of the United States Centers for Disease Control and Prevention.

## Conflict of interest

RE-S is an Editorial Board Member for *Current Developments in Nutrition* and played no role in the Journal’s evaluation of the manuscript. All other authors report no conflicts of interest.
