# Supplementary material for: Micronutrients Associated With Anemia in School-age Children and Adolescents 2005–2018: Biomarkers Reflecting Inflammation and Nutritional Determinants of Anemia (BRINDA) Project
Source: Curr Dev Nutr. 2025 Jul 5;9(8):107502. doi: 10.1016/j.cdnut.2025.107502 (PMC12362516; doi:10.1016/j.cdnut.2025.107502)
Supplement: Multimedia component 1 [file mmc1.pdf]

BRINDA
Biomarkers Reflecting Inflammation and Nutritional Determinants of Anemia

Micronutrients associated with anemia in school-age children and adolescents 2005–2018: BRINDA Project

Werner et al 2025

2024-12-06

Contents

List of Supplementary Figures

1 Conceptual framework for anemia in school-age children and adolescents 8
2 Iron deficiency by sex in children age (A) 5–9 y, (B) 10–14 y, and (C) 15–19 y in the BRINDA Project 9

List of Supplementary Tables

1 Anemia in school-age children and adolescents 5–19 y: BRINDA Project 2
2 Prevalence of inflammation and micronutrient deficiencies in school-age children and adolescents 5–19 y: BRINDA Project 3
3 Prevalence ratios for bivariate associations between anemia and sex, age, and socioeconomic status in school-age children and adolescents 5–19 y: BRINDA Project. 4
4 Population attributable fractions for the burden of anemia in school-age children and adolescents 5–19 y: BRINDA Project 5
5 Prevalence ratios for the association of BMI category with anemia in school-age children and adolescents 5–19 y: BRINDA Project 6
6 Prevalence ratios of anemia assessed by multivariate modified Poisson regression models in school-age children and adolescents 5–19 y: BRINDA Project 7

Supplementary Table 1. Anemia in school-age children and adolescents 5–19 y: BRINDA Project

Country survey	Age range, y	*n*	Any anemia, %	Anemia classification[1]		
				Mild, %	Moderate, %	Severe, %
Azerbaijan (2013)	15–19	361	35.2 (29.9, 40.7)	19.9 (15.6, 24.8)	13.6 (10.1, 17.6)	1.7 (0.7, 3.3)
Bangladesh (2012)	5–19	1432	16.1 (13.6, 18.7)	10.2 (8.5, 12.1)	5.9 (4.6, 7.3)	0
Colombia (2010)	5–19	11,281	5.7 (5.2, 6.2)	2.2 (2.0, 2.5)	3.4 (3.1, 3.8)	0.0 (0.0, 0.1)
Côte d'Ivoire (2007)	15–19	110	59.1 (48.5, 69.2)	30.0 (21.7, 39.3)	28.2 (19.1, 38.7)	0.9 (0.0, 4.1)
Ecuador (2012)	5–19	7362	4.8 (4.2, 5.5)	3.3 (2.9, 3.8)	1.4 (1.0, 1.8)	0.1 (0.0, 0.2)
Georgia (2009)	15–19	178	25.3 (18.4, 33.1)	16.9 (11.3, 23.7)	7.9 (4.3, 12.8)	0.6 (0.0, 2.5)
Laos (2006)	15–19	170	40.6 (33.4, 48.1)	18.2 (13.5, 23.7)	20.6 (14.2, 28.1)	1.8 (0.4, 4.5)
Liberia (2011)	15–19	378	39.4 (33.7, 45.3)	25.1 (20.9, 29.7)	14.0 (10.7, 17.8)	0.3 (0.0, 1.2)
Malawi (2016)	5–19	918	23.2 (19.5, 27.2)	11.4 (9.1, 14.1)	10.7 (8.5, 13.1)	1.1 (0.5, 2.0)
Mexico (2006)	5–19	4323	12.6 (11.6, 13.7)	6.4 (5.7, 7.2)	6.2 (5.4, 7.0)	0
Mexico (2012)	5–14	3786	9.5 (8.6, 10.5)	4.6 (3.9, 5.3)	4.8 (4.2, 5.6)	0.1 (0.0, 0.2)
Nepal (2016)	10–19	2861	15.6 (13.5, 17.8)	11.1 (9.6, 12.9)	4.3 (3.5, 5.3)	0.1 (0.0, 0.2)
Pakistan (2011)	15–19	112	47.3 (38.1, 56.6)	25.0 (17.4, 33.8)	18.8 (12.2, 26.8)	3.6 (1.1, 8.3)
Papua New Guinea (2005)	15–19	132	40.9 (32.6, 49.6)	17.4 (12.3, 23.4)	21.2 (14.7, 28.9)	2.3 (0.5, 6.0)
United Kingdom (2014)	5–19	776	4.8 (3.5, 6.3)	4.0 (2.8, 5.5)	0.6 (0.2, 1.4)	0.1 (0.0, 0.6)
United States (2018)	5–19	20,163	4.8 (4.3, 5.3)	3.6 (3.3, 4.0)	1.1 (0.9, 1.3)	0.0 (0.0, 0.1)
Vietnam (2010)	15–19	191	6.8 (3.8, 10.9)	3.7 (1.7, 6.6)	2.6 (0.9, 5.7)	0.5 (0.0, 2.4)
**Median (range)[2]**	–	–	**16.1 (4.8–59.1)**	**11.1 (2.2–30.0)**	**6.2 (0.6–28.2)**	**0.3 (0.0–3.6)**

Values are mean (95% CI). BRINDA, Biomarkers Reflecting Inflammation and Nutritional Determinants of Anemia; CI, confidence interval.

[1]Any anemia was assessed using WHO cutoff values for altitude and smoking-adjusted hemoglobin (children <12y: <115g/L; children 12-14y: <120g/L; females ≥15 y: <120 g/L; males ≥15 y: <130 g/L) and further classified as severe if <80 g/L, moderate if ≥80 g/L and <110 g/L, or mild for all remaining cases of anemia.

[2]Median (range) reports the unweighted median, lowest, and highest prevalence across surveys.

Supplementary Table 2. Prevalence of inflammation and micronutrient deficiencies in school-age children and adolescents 5–19 y: BRINDA Project

		Inflammation		Iron deficiency		VA deficiency		Folate deficiency		Vit B_{12} deficiency		Zinc deficiency	
**Country survey**	**Age range, y**	***n***	**% (95% CI)**	***n***	**% (95% CI)**	***n***	**% (95% CI)**	***n***	**% (95% CI)**	***n***	**% (95% CI)**	***n***	**% (95% CI)**
Azerbaijan (2013)	15–19	361	20.5 (16.4, 25.0)	361	38.2 (33.0, 43.6)	361	1.1 (0.2, 3.0)	345	38.3 (32.9, 43.8)	173	22.0 (15.7, 29.2)	_	_
Bangladesh (2012)	5–19	1432	13.8 (11.9, 15.8)	1417	7.1 (5.8, 8.5)	1421	19.2 (16.7, 21.9)	160	42.5 (34.4, 50.9)	164	2.4 (0.8, 5.6)	146	21.2 (15.1, 28.3)
Colombia (2010)	5–19	11,281	13.6 (12.8, 14.4)	11,281	13.8 (13.1, 14.5)	_	_	_	_	7008	3.8 (3.3, 4.2)	_	_
Côte d'Ivoire (2007)	15–19	110	34.5 (26.4, 43.3)	110	16.4 (10.8, 23.2)	110	0	106	87.7 (79.5, 93.7)	56	19.6 (10.3, 32.0)	_	_
Ecuador (2012)	5–19	7362	6.8 (6.2, 7.4)	7361	5.7 (5.2, 6.3)	3281	10.5 (8.9, 12.2)	7361	0.4 (0.3, 0.5)	4063	1.0 (0.5, 1.6)	7341	33.7 (32.3, 35.2)
Georgia (2009)	15–19	178	12.4 (7.7, 18.3)	178	0	_	_	26	38.5 (20.1, 59.4)	_	_	_	_
Laos (2006)	15–19	170	12.9 (8.8, 18.0)	170	42.9 (34.4, 51.8)	_	_	_	_	_	_	_	_
Liberia (2011)	15–19	378	18.0 (14.1, 22.4)	378	38.6 (33.4, 44.0)	378	1.3 (0.4, 3.2)	_	_	_	_	_	_
Malawi (2016)	5–19	918	31.9 (28.3, 35.7)	918	7.0 (5.3, 8.9)	918	3.3 (2.1, 4.7)	160	18.8 (12.8, 25.9)	160	11.2 (6.5, 17.7)	912	57.6 (51.3, 63.6)
Mexico (2006)	5–19	4323	9.9 (9.0, 10.8)	4323	26.4 (24.9, 27.9)	_	_	_	_	_	_	2940	16.2 (14.4, 18.0)
Mexico (2012)	5–14	3786	8.8 (7.9, 9.8)	3785	14.4 (13.3, 15.6)	2773	1.6 (1.1, 2.0)	3783	0.1 (0.0, 0.2)	3784	0.4 (0.2, 0.7)	_	_
Nepal (2016)	10–19	2861	6.6 (5.6, 7.7)	2861	15.2 (13.5, 16.9)	2861	2.2 (1.6, 2.9)	1838	24.4 (21.5, 27.4)	_	_	_	_
Pakistan (2011)	15–19	112	19.6 (13.2, 27.4)	99	42.4 (32.9, 52.4)	84	40.5 (30.6, 50.9)	96	61.5 (51.2, 71.1)	97	55.7 (45.8, 65.2)	79	40.5 (29.7, 52.0)
Papua New Guinea (2005)	15–19	132	31.1 (22.7, 40.4)	132	15.2 (9.3, 22.5)	132	0	_	_	_	_	_	_
United Kingdom (2014)	5–19	776	5.3 (3.8, 7.1)	767	20.3 (17.4, 23.5)	729	1.0 (0.4, 1.8)	_	_	748	2.7 (1.7, 4.0)	631	4.3 (2.9, 6.1)
United States (2018)	5–19	20,163	7.4 (7.1, 7.8)	11,847	14.2 (13.4, 15.0)	13,845	0.4 (0.3, 0.5)	19408	0.2 (0.2, 0.3)	12,515	0.6 (0.4, 0.7)	667	4.8 (3.2, 6.9)
Vietnam (2010)	15–19	191	4.7 (2.3, 8.4)	191	16.2 (11.6, 21.7)	184	2.2 (0.7, 4.8)	177	13.0 (8.3, 18.9)	52	5.8 (0.9, 17.5)	191	38.7 (31.0, 46.9)
**Median (range)[1]**	–	–	**12.9 (4.7–34.5)**	–	**15.2 (0–42.9)**	–	**1.6 (0–40.5)**	–	**24.4 (0.1–87.7)**	–	**3.8 (0.4–55.7)**	–	**27.5 (4.3–57.6)**

Cutoff values for inflammation (CRP >5 mg/L and/or AGP >1 g/L) and inflammation-adjusted micronutrient deficiencies: iron (ferritin <12 µg/L or sTfR >8.3 mg/L [Papua New Guinea]); vitamin A (retinol <0.7 µmol/L or RBP <0.7 µmol/L [Azerbaijan, Côte d'Ivoire, Liberia, Malawi, Nepal, and Papua New Guinea]); folate (serum folate <10 nmol/L or RBC folate <340 nmol/L [Nepal]); vitamin B_{12} (serum vitamin B_{12} <150 pmol/L); zinc (IZiNCG 2012). BRINDA inflammation-adjustment method applied for iron (children 5–19 y) and vitamin A (children 5–14 y).

AGP, α-1-acid glycoprotein; BRINDA, Biomarkers Reflecting Inflammation and Nutritional Determinants of Anemia; CI, confidence interval; CRP, C-reactive protein; IZiNCG, International Zinc Nutrition Consultative Group; RBC, red blood cell; RBP, retinol binding protein; sTfR, soluble transferrin receptor; VA, vitamin A

[1]Median (range) reports the unweighted median, lowest, and highest prevalence across surveys.

Supplementary Table 3. Prevalence ratios for bivariate associations between anemia and sex, age, and socioeconomic status in school-age children and adolescents 5–19 y: BRINDA Project

			SES category			
**Country survey**	**Female**	**Age, y**	**Low**	**Medium**	**High**	***P*-value**
**Children 5–9 y**						
Bangladesh (2012)	0.96 (0.68, 1.37)	0.92 (0.79, 1.08)	1.35 (0.80, 2.27)	0.74 (0.42, 1.33)	REF	0.032
Colombia (2010)	1.03 (0.82, 1.30)	0.86 (0.79, 0.93)***	2.23 (1.10, 4.53)**	1.23 (0.59, 2.60)	REF	<0.001
Ecuador (2012)	0.65 (0.45, 0.92)**	0.79 (0.69, 0.89)***	1.55 (0.83, 2.91)	0.94 (0.48, 1.86)	REF	0.035
Malawi (2016)	0.87 (0.64, 1.20)	0.87 (0.78, 0.97)**	2.84 (1.35, 5.95)***	2.05 (0.97, 4.34)*	REF	0.014
Mexico (2006)	1.05 (0.87, 1.27)	0.86 (0.79, 0.93)***	1.71 (1.04, 2.79)**	1.55 (0.93, 2.58)*	REF	0.091
Mexico (2012)	1.11 (0.89, 1.38)	0.80 (0.74, 0.87)***	1.22 (0.82, 1.81)	1.25 (0.83, 1.88)	REF	0.555
United Kingdom (2014)	0.32 (0.07, 1.52)	0.55 (0.37, 0.81)***	1.81 (0.30, 10.82)	2.59 (0.47, 14.13)	REF	0.547
United States (2018)	1.11 (0.81, 1.52)	0.82 (0.73, 0.92)***	1.39 (0.80, 2.40)	1.12 (0.63, 1.99)	REF	0.327
**Children 10–14 y**						
Bangladesh (2012)	1.25 (0.87, 1.80)	1.07 (0.93, 1.22)	1.75 (0.94, 3.27)*	1.68 (0.90, 3.12)	REF	0.191
Colombia (2010)	1.01 (0.75, 1.35)	1.14 (1.04, 1.26)***	3.10 (1.37, 7.00)***	1.57 (0.66, 3.73)	REF	<0.001
Ecuador (2012)	1.35 (0.85, 2.14)	1.66 (1.37, 2.00)***	0.66 (0.35, 1.24)	0.74 (0.39, 1.42)	REF	0.436
Malawi (2016)	0.71 (0.44, 1.17)	1.07 (0.90, 1.29)	2.16 (0.88, 5.29)*	1.93 (0.80, 4.67)	REF	0.238
Mexico (2006)	1.55 (0.96, 2.51)*	1.14 (1.00, 1.31)*	1.07 (0.53, 2.16)	1.09 (0.53, 2.26)	REF	0.973
Mexico (2012)	1.04 (0.67, 1.61)	1.07 (0.73, 1.56)	1.71 (0.77, 3.80)	1.17 (0.51, 2.69)	REF	0.198
Nepal (2016)	2.09 (1.52, 2.86)***	1.13 (1.03, 1.24)***	0.78 (0.53, 1.15)	0.93 (0.62, 1.39)	REF	0.329
United Kingdom (2014)	9.19 (1.16, 72.87)**	1.57 (0.90, 2.74)	1.33 (0.24, 7.25)	0.44 (0.04, 4.82)	REF	0.609
United States (2018)	3.12 (2.37, 4.12)***	1.34 (1.23, 1.46)***	2.43 (1.51, 3.93)***	1.80 (1.10, 2.96)**	REF	<0.001
**Children 15–19 y**						
Azerbaijan (2013)	–	1.02 (0.92, 1.13)	1.21 (0.74, 1.96)	0.89 (0.54, 1.50)	REF	0.313
Bangladesh (2012)	–	0.90 (0.72, 1.14)	0.85 (0.33, 2.15)	0.68 (0.27, 1.71)	REF	0.705
Colombia (2010)	–	1.02 (0.92, 1.13)	1.29 (0.78, 2.15)	0.82 (0.47, 1.44)	REF	0.032
Côte d'Ivoire (2007)	–	1.14 (1.00, 1.30)**	1.32 (0.60, 2.90)	1.13 (0.52, 2.47)	REF	0.744
Ecuador (2012)	–	0.99 (0.90, 1.10)	1.95 (1.09, 3.51)**	1.71 (0.94, 3.12)*	REF	0.079
Georgia (2009)	–	0.95 (0.79, 1.15)	–	–	–	–
Laos (2006)	–	1.06 (0.93, 1.21)	1.84 (0.85, 3.96)	1.31 (0.59, 2.89)	REF	0.200
Liberia (2011)	–	0.98 (0.90, 1.07)	1.35 (0.86, 2.14)	1.49 (1.01, 2.20)**	REF	0.134
Malawi (2016)	–	1.07 (0.90, 1.28)	0.67 (0.31, 1.47)	0.81 (0.40, 1.64)	REF	0.609
Mexico (2006)	–	1.07 (0.92, 1.24)	0.83 (0.46, 1.49)	0.75 (0.40, 1.39)	REF	0.656
Nepal (2016)	2.14 (1.58, 2.90)***	0.93 (0.86, 1.01)	1.13 (0.77, 1.66)	1.37 (0.93, 2.00)	REF	0.190
Pakistan (2011)	–	0.97 (0.75, 1.24)	1.27 (0.52, 3.14)	1.54 (0.63, 3.75)	REF	0.593
Papua New Guinea (2005)	–	0.99 (0.86, 1.15)	1.51 (0.72, 3.19)	1.42 (0.67, 3.01)	REF	0.540
United Kingdom (2014)	–	0.95 (0.63, 1.43)	2.33 (0.49, 10.97)	2.51 (0.51, 12.43)	REF	0.506
United States (2018)	9.12 (6.96, 11.96)***	1.04 (0.99, 1.11)	1.86 (1.37, 2.53)***	1.61 (1.17, 2.21)***	REF	<0.001
Vietnam (2010)	–	0.83 (0.59, 1.17)	–	–	–	–

Variables associated with anemia with $P < 0.1$ were included in multivariable models. SES categories were defined by classifying the highest quintile as "high," the lowest two quintiles as "low," and the third and fourth quintile as "medium", and *P*-values for Wald statistics determined overall significance of the SES variable.

BRINDA, Biomarkers Reflecting Inflammation and Nutritional Determinants of Anemia; REF, reference; SES, socioeconomic status.

*$P < 0.1$ **$P < 0.05$ ***$P < 0.01$.

Supplementary Table 4. Population attributable fractions for the burden of anemia in school-age children and adolescents 5–19 y: BRINDA Project

Country survey	Inflammation, %	Iron def, %	Vit A def, %	Folate def, %	Vit B_{12} def, %	Zinc def, %
**Children 5–9 y**						
Bangladesh (2012)	–	3.6*	–	–	–	–
Colombia (2010)	5.2*	12.9***	–	–	1.9	–
Ecuador (2012)	6.7**	4.1**	7.5**	–	–	9.7*
Malawi (2016)	35.6***	4.0*	–	–	–	13.9
Mexico (2006)	–	3.2	–	–	–	–
Mexico (2012)	–	1.4	2.5**	–	–	–
United Kingdom (2014)	–	31.6	–	–	–	–
United States (2018)	6.0**	–	–	–	–	–
**Children 10–14 y**						
Bangladesh (2012)	–	6.8**	15.7***	–	–	–
Colombia (2010)	5.4	12.3***	–	–	4.1*	–
Ecuador (2012)	–	18.4***	–	–	2.3	16.8
Malawi (2016)	12.6	–	–	–	–	–
Mexico (2006)	5.8*	–	–	–	–	–
Mexico (2012)	–	–	–	–	–	–
Nepal (2016)	–	12.0***	5.5***	–	–	–
United Kingdom (2014)	–	40.5**	–	–	–	–
United States (2018)	8.1***	28.1***	0.5	–	–	–
**Children 15–19 y**						
Azerbaijan (2013)	–	39.2***	–	7.1	–	–
Bangladesh (2012)	–	22.9**	–	37.0*	–	–
Colombia (2010)	8.1**	27.7***	–	–	–	–
Côte d'Ivoire (2007)	14.0*	8.5**	–	–	–	–
Ecuador (2012)	2.1	52.2***	–	–	–	18.2*
Georgia (2009)	8.7	–	–	–	–	–
Laos (2006)	–	13.7	–	–	–	–
Liberia (2011)	10.6***	32.7***	–	–	–	–
Malawi (2016)	–	17.9**	1.8	–	-9.6	–
Mexico (2006)	7.6*	37.7***	–	–	–	–
Nepal (2016)	4.8**	21.7***	1.7**	–	–	–
Pakistan (2011)	–	–	–	–	–	–
Papua New Guinea (2005)	–	17.1***	–	–	–	–
United Kingdom (2014)	–	44.9**	–	–	–	–
United States (2018)	4.7**	35.0***	0.6***	–	0.6	–
Vietnam (2010)	–	68.1***	–	18.9*	–	52.4*

Population attributable fractions were estimated from PR presented in Table 3 and PE for inflammation and micronutrient deficiencies presented in Table 1 as follows: (PE x (PR – 1)) / (PE x (PR – 1) + 1). Population attributable fractions controlled for other micronutrients, age, sex, and socioeconomic status as indicated and are not recommended to be summed across factors. Cutoff values for inflammation (CRP >5 mg/L and/or AGP >1 g/L) and inflammation-adjusted micronutrient deficiencies: iron (ferritin <12 µg/L or sTfR >8.3 mg/L [Papua New Guinea]); vitamin A (retinol <0.7 µmol/L or RBP <0.7 µmol/L [Azerbaijan, Côte d'Ivoire, Liberia, Malawi, Nepal, and Papua New Guinea]); folate (serum folate <10 nmol/L or RBC folate <340 nmol/L [Nepal]); vitamin B_{12} (serum vitamin B_{12} <150 pmol/L); zinc (IZiNCG 2012). BRINDA inflammation-adjustment method applied for iron (children 5–19 y) and vitamin A (children 5–14 y).

AGP, α-1-acid glycoprotein; BRINDA, Biomarkers Reflecting Inflammation and Nutritional Determinants of Anemia; CRP, C-reactive protein; Def, deficiency; IZiNCG, International Zinc Nutrition Consultative Group; PE, prevalence of exposure; PR, prevalence ratio; RBC, red blood cell; RBP, retinol binding protein; sTfR, soluble transferrin receptor; Vit, vitamin.

$^{*}P < 0.05$, $^{**}P < 0.01$, $^{***}P < 0.001$.

Supplementary Table 5. Prevalence ratios for the association of BMI category with anemia in school-age children and adolescents 5–19 y: BRINDA Project

	Thin		Normal		Overweight		Obese		Overall
**Country survey**	***n***	**PR (95% CI)**	***n***	**PR (95% CI)**	***n***	**PR (95% CI)**	***n***	**PR (95% CI)**	***P*-value**
**Children 5–9 y**									
Colombia (2010)	59	0.26 (0.04, 1.84)	3453	REF	485	0.82 (0.54, 1.22)	167	0.27 (0.09, 0.85)**	0.057
Ecuador (2012)	37	1.93 (0.61, 6.10)	2241	REF	570	0.54 (0.30, 0.97)**	291	0.82 (0.43, 1.57)	0.112
Malawi (2016)	14	1.02 (0.37, 2.76)	359	REF	22	0.48 (0.15, 1.53)	3	–	0.675
Mexico (2006)	35	0.92 (0.38, 2.22)	1689	REF	428	0.89 (0.67, 1.17)	252	0.59 (0.38, 0.90)**	0.096
Mexico (2012)	31	0.57 (0.14, 2.31)	1770	REF	506	0.86 (0.63, 1.18)	312	0.77 (0.51, 1.15)	0.442
United Kingdom (2014)	1	–	127	REF	38	–	36	0.59 (0.07, 4.88)	0.970
United States (2018)	30	1.18 (0.16, 8.47)	2362	REF	843	0.88 (0.54, 1.43)	844	0.63 (0.36, 1.10)	0.427
**Children 10–14 y**									
Colombia (2010)	105	1.12 (0.46, 2.74)	3347	REF	618	1.30 (0.89, 1.89)	170	0.97 (0.45, 2.07)	0.590
Ecuador (2012)	37	2.05 (0.50, 8.42)	2012	REF	581	0.59 (0.29, 1.19)	252	0.90 (0.39, 2.10)	0.333
Malawi (2016)	30	1.26 (0.54, 2.93)	308	REF	1	–	_	–	0.869
Mexico (2006)	13	0.91 (0.13, 6.53)	718	REF	316	1.08 (0.69, 1.68)	177	0.60 (0.30, 1.21)	0.477
Mexico (2012)	22	–	639	REF	245	0.71 (0.40, 1.26)	192	0.24 (0.09, 0.67)***	0.042
Nepal (2016)	259	0.94 (0.64, 1.37)	1242	REF	65	0.58 (0.24, 1.42)	12	1.26 (0.31, 5.09)	0.656
United Kingdom (2014)	4	–	191	REF	88	0.62 (0.13, 2.99)	43	–	0.949
United States (2018)	66	2.43 (0.99, 5.96)*	3398	REF	1711	1.50 (1.12, 2.00)***	1799	1.25 (0.92, 1.69)	0.019
**Children 15–19 y**									
Azerbaijan (2013)	7	–	276	REF	61	0.64 (0.37, 1.10)	16	0.98 (0.43, 2.22)	0.455
Colombia (2010)	36	1.63 (0.60, 4.39)	2049	REF	434	0.54 (0.32, 0.91)**	98	1.34 (0.69, 2.64)	0.054
Côte d'Ivoire (2007)	2	–	98	REF	6	1.41 (0.56, 3.51)	2	0.84 (0.12, 6.10)	0.901
Ecuador (2012)	9	1.77 (0.44, 7.17)	893	REF	265	0.96 (0.65, 1.43)	90	0.80 (0.40, 1.57)	0.769
Georgia (2009)	1	–	149	REF	22	0.33 (0.08, 1.37)	5	1.45 (0.35, 6.01)	0.445
Laos (2006)	1	–	161	REF	7	0.70 (0.17, 2.85)	1	–	0.788
Malawi (2016)	5	0.76 (0.10, 5.51)	140	REF	20	0.95 (0.37, 2.41)	_	–	0.958
Mexico (2006)	12	0.59 (0.08, 4.23)	429	REF	153	0.87 (0.52, 1.46)	63	0.78 (0.36, 1.71)	0.851
Nepal (2016)	93	1.61 (1.08, 2.40)**	1107	REF	54	0.82 (0.40, 1.66)	4	–	0.119
Pakistan (2011)	7	0.57 (0.14, 2.35)	96	REF	7	0.86 (0.27, 2.75)	1	–	0.885
Papua New Guinea (2005)	1	–	102	REF	28	0.29 (0.11, 0.81)**	1	–	0.137
United Kingdom (2014)	_	–	141	REF	37	0.25 (0.03, 1.92)	19	0.49 (0.07, 3.75)	0.349
United States (2018)	72	1.18 (0.49, 2.85)	4341	REF	1693	1.19 (0.96, 1.48)	1614	1.46 (1.19, 1.80)***	0.004
Vietnam (2010)	16	0.91 (0.12, 6.97)	174	REF	_	–	_	–	0.925

Bivariate associations between anemia and BMI category with $P < 0.1$ were included in multivariable models in Supplementary Table 6. BMI categories based on the following WHO *z*-scores for BMI-for-age: thin ≤−2; normal >−2 & <1; overweight ≥1 & <2; obese ≥2. Anthropometry was not assessed for children in Bangladesh 2012 or Liberia 2011 surveys. In the UK, none of the children aged 5–9 y with overweight or children aged 10–14 y with obesity had anemia. In Mexico 2012, none of the children 10–14y with thinness had anemia.

BRINDA, Biomarkers Reflecting Inflammation and Nutritional Determinants of Anemia; PR, prevalence ratio; REF, reference group.

**P*<0.1 **P*<0.05 ****P*<0.01

Supplementary Table 6. Prevalence ratios of anemia assessed by multivariate modified Poisson regression models in school-age children and adolescents 5–19 y: BRINDA Project

				SES category			BMI category				Inflammation and micronutrient deficiencies			
**Country survey**	***n***	**Female**	**Age, y**	**Medium**	**Low**	***P*-value**	**Thin**	**Overweight**	**Obese**	***P*-value**	**Inflammation**	**Iron**	**Vitamin A**	**Vitamin B_{12}**
**Children 5–9 y**														
Colombia (2010)	3882	–	0.91 (0.83, 0.99)*	1.20 (0.54, 2.65)	1.98 (0.93, 4.23)	0.003	–	0.83 (0.54, 1.27)	0.35 (0.11, 1.09)	0.147	1.43 (1.05, 1.94)*	2.71 (2.01, 3.64)***	–	1.66 (1.01, 2.73)*
Mexico (2006)	2403	–	0.88 (0.82, 0.95)**	1.49 (0.92, 2.42)	1.53 (0.96, 2.45)	0.241	0.90 (0.40, 2.03)	0.92 (0.71, 1.19)	0.65 (0.43, 0.98)*	0.270	–	1.13 (0.91, 1.39)	–	–
**Children 10–14 y**														
Mexico (2012)	1076	–	–	–	–	–	–	0.71 (0.41, 1.23)	0.24 (0.09, 0.66)**	0.017	–	–	–	–
United States (2018)	3156	2.98 (1.85, 4.80)***	1.18 (1.04, 1.34)*	–	2.50 (1.28, 4.88)**	0.025	1.60 (0.25, 10.20)	1.58 (1.09, 2.28)*	1.25 (0.84, 1.87)	0.141	2.40 (1.56, 3.71)***	3.94 (2.85, 5.46)***	4.36 (0.62, 30.61)	–
**Children 15–19 y**														
Colombia (2010)	2617	–	–	0.92 (0.52, 1.65)	1.50 (0.88, 2.55)	0.017	1.65 (0.65, 4.16)	0.56 (0.34, 0.92)*	1.27 (0.67, 2.40)	0.077	1.47 (1.03, 2.10)*	2.70 (2.02, 3.60)***	–	–
United States (2018)	3437	8.06 (4.24, 15.30)***	–	1.76 (1.15, 2.69)**	1.91 (1.27, 2.87)**	0.018	1.03 (0.35, 3.04)	1.12 (0.86, 1.46)	1.58 (1.22, 2.03)***	0.022	1.20 (0.90, 1.60)	4.01 (3.22, 5.00)***	3.93 (2.83, 5.46)***	1.55 (0.83, 2.89)

Surveys and age groups with bivariate associations between anemia and BMI category ($P < 0.1$) are shown for comparison to principle models (Table 3). SES categories were defined by classifying the lowest two quintiles as "low," the third and fourth quintile as "medium," and the highest quintile as the referent group. Cutoff values for inflammation (CRP >5 mg/L and/or AGP >1 g/L) and inflammation-adjusted micronutrient deficiencies: iron (ferritin <12 µg/L or sTfR >8.3 mg/L [Papua New Guinea]); vitamin A (retinol <0.7 µmol/L or RBP <0.7 µmol/L [Azerbaijan, Côte d'Ivoire, Liberia, Malawi, Nepal, and Papua New Guinea]); folate (serum folate <10 nmol/L or RBC folate <340 nmol/L [Nepal]); vitamin B_{12} (serum vitamin B_{12} <150 pmol/L); zinc (IZiNCG 2012). BRINDA inflammation-adjustment method applied for iron (children 5–19 y) and vitamin A (children 5–14 y). Multivariable models adjusted for sex, age, SES, and BMI category when *P* values for bivariate associations with anemia were <0.1. Inflammation and micronutrient biomarkers were included in models when *P* values for bivariate associations with anemia were <0.1. Reference categories were high SES and normal BMI. Thin BMI was removed from Colombia 5–9 y and Mexico (2012) 10–14 y models because none of these children had anemia.

AGP, α-1-acid glycoprotein; BMI, body mass index; BRINDA, Biomarkers Reflecting Inflammation and Nutritional Determinants of Anemia; CRP, C-reactive protein; IZiNCG, International Zinc Nutrition Consultative Group; PR, prevalence ratio; RBC, red blood cell; RBP, retinol binding protein; SES, socioeconomic status; sTfR, soluble transferrin receptor

$^{*}P < 0.05$, $^{**}P < 0.01$, $^{***}P < 0.001$

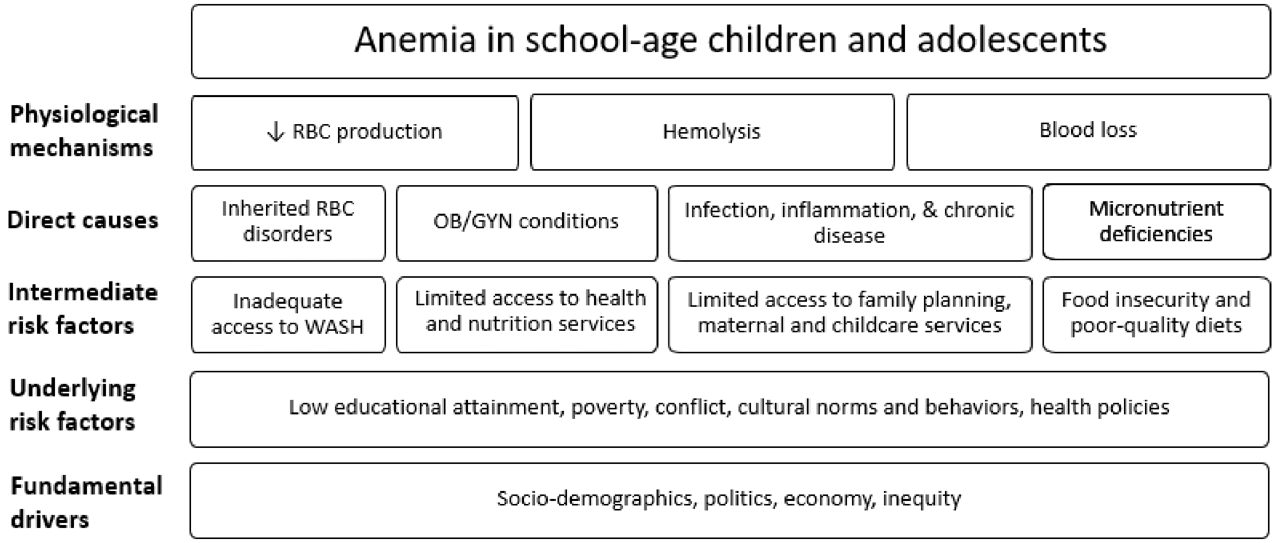

Supplementary Figure 1. Conceptual framework for anemia in school-age children and adolescents.

Framework adapted from World Health Organization (WHO) 2023 *Accelerating anaemia reduction: a comprehensive framework for action.*

OB/GYN, obstetrics and gynecology; RBC, red blood cell; WASH, water sanitation, and hygiene.

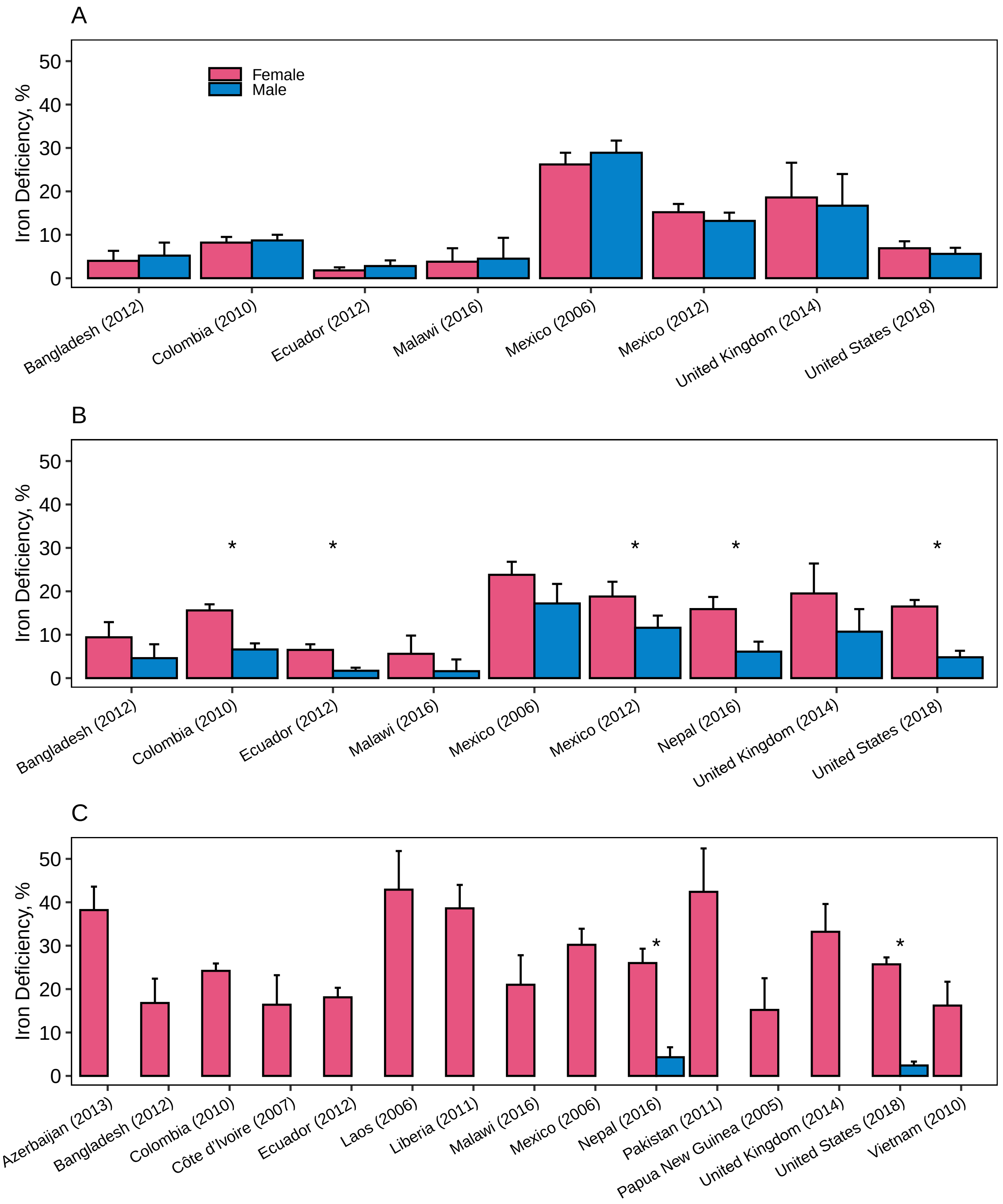

Supplementary Figure 2. Iron deficiency by sex in children age (A) 5–9 y, (B) 10–14 y, and (C) 15–19 y in the BRINDA Project.

BRINDA inflammation-adjusted iron deficiency was assessed using ferritin <15umol/L or sTfR >8.3mg/L when ferritin was unavailable.

BRINDA, Biomarkers Reflecting Inflammation and Nutritional Determinants of Anemia.

**P*<0.05
